# *KCNV2-*Associated Retinopathy: Genetics, Electrophysiology, and Clinical Course—*KCNV2* Study Group Report 1

**DOI:** 10.1016/j.ajo.2020.11.022

**Published:** 2021-05

**Authors:** Michalis Georgiou, Anthony G. Robson, Kaoru Fujinami, Shaun M. Leo, Ajoy Vincent, Fadi Nasser, Thales Antônio Cabral De Guimarães, Samer Khateb, Nikolas Pontikos, Yu Fujinami-Yokokawa, Xiao Liu, Kazushige Tsunoda, Takaaki Hayashi, Mauricio E. Vargas, Alberta A.H.J. Thiadens, Emanuel R. de Carvalho, Xuan-Thanh-An Nguyen, Gavin Arno, Omar A. Mahroo, Maria Inmaculada Martin-Merida, Belen Jimenez-Rolando, Gema Gordo, Ester Carreño, Ayuso Carmen, Dror Sharon, Susanne Kohl, Rachel M. Huckfeldt, Bernd Wissinger, Camiel J.F. Boon, Eyal Banin, Mark E. Pennesi, Arif O. Khan, Andrew R. Webster, Eberhart Zrenner, Elise Héon, Michel Michaelides

**Affiliations:** a Moorfields Eye Hospital, London, United Kingdom; b University College London Institute of Ophthalmology, London, United Kingdom; c Laboratory of Visual Physiology, Division of Vision Research, National Institute of Sensory Organs, National Hospital Organization Tokyo Medical Center, Tokyo, Japan; d Department of Ophthalmology, Keio University School of Medicine, Tokyo, Ontario, Japan; e Department of Ophthalmology and Vision Sciences, The Hospital for Sick Children, University of Toronto, Toronto, Ontario, Canada; f Institute for Ophthalmic Research, Centre for Ophthalmology, University of Tübingen, Tübingen, Germany; g Department of Ophthalmology, Hadassah Medical Center, Faculty of Medicine, The Hebrew University of Jerusalem, Jerusalem, Israel; h Department of Health Policy and Management, Keio University School of Medicine, Tokyo, Japan; i Department of Ophthalmology, Katsushika Medical Center, The Jikei University School of Medicine, Tokyo, Japan; j Department of Ophthalmology, Oregon Health and Science University, Casey Eye Institute, Portland, Oregon, USA; k Department of Ophthalmology, Erasmus Medical Center, Rotterdam, the Netherlands; l Department of Ophthalmology, Amsterdam UMC, Academic Medical Center, Amsterdam, the Netherlands; m Department of Ophthalmology, Leiden University Medical Center, Leiden, the Netherlands; n Department of Genetics, Instituto de Investigación Sanitaria-Fundación Jiménez Díaz University Hospital-Universidad Autónoma de Madrid, Madrid, Spain; o Center for Biomedical Network Research on Rare Diseases, Instituto de Salud Carlos III, Madrid, Spain; p Department of Ophthalmology, Instituto de Investigación Sanitaria-Fundación Jiménez Díaz University Hospital-Universidad Autónoma de Madrid, Madrid, Spain; q Department of Ophthalmology, Massachusetts Eye and Ear Infirmary, Harvard Medical School, Boston, Massachusetts, USA; r Department of Ophthalmology, Cleveland Clinic Lerner College of Medicine of Case Western University, Cleveland, Ohio, USA; s Eye Institute, Cleveland Clinic Abu Dhabi, Abu Dhabi, United Arab Emirates

## Abstract

**Purpose:**

To investigate genetics, electrophysiology, and clinical course of *KCNV2-*associated retinopathy in a cohort of children and adults.

**Study design:**

This was a multicenter international clinical cohort study.

**Methods:**

Review of clinical notes and molecular genetic testing. Full-field electroretinography (ERG) recordings, incorporating the international standards, were reviewed and quantified and compared with age and recordings from control subjects.

**Results:**

In total, 230 disease-associated alleles were identified from 117 patients, corresponding to 75 different *KCNV2* variants, with 28 being novel. The mean age of onset was 3.9 years old. All patients were symptomatic before 12 years of age (range, 0-11 years). Decreased visual acuity was present in all patients, and 4 other symptoms were common: reduced color vision (78.6%), photophobia (53.5%), nyctalopia (43.6%), and nystagmus (38.6%). After a mean follow-up of 8.4 years, the mean best-corrected visual acuity (BCVA ± SD) decreased from 0.81 ± 0.27 to 0.90 ± 0.31 logarithm of minimal angle of resolution. Full-field ERGs showed pathognomonic waveform features. Quantitative assessment revealed a wide range of ERG amplitudes and peak times, with a mean rate of age-associated reduction indistinguishable from the control group. Mean amplitude reductions for the dark-adapted 0.01 ERG, dark-adapted 10 ERG a-wave, and LA 3.0 30 Hz and LA3 ERG b-waves were 55%, 21%, 48%, and 74%, respectively compared with control values. Peak times showed stability across 6 decades.

**Conclusion:**

In *KCNV2*-associated retinopathy, full-field ERGs are diagnostic and consistent with largely stable peripheral retinal dysfunction. Report 1 highlights the severity of the clinical phenotype and established a large cohort of patients, emphasizing the unmet need for trials of novel therapeutics.

## Highlights

• The current study established the largest and most characterized cohort of molecularly confirmed patients with *KCNV2*-associated retinopathy.
• Report 1 highlights the genetic background, evidence of electroretinography stability over a broad age range, and the severe phenotype of the disease.

**Figure 1:**
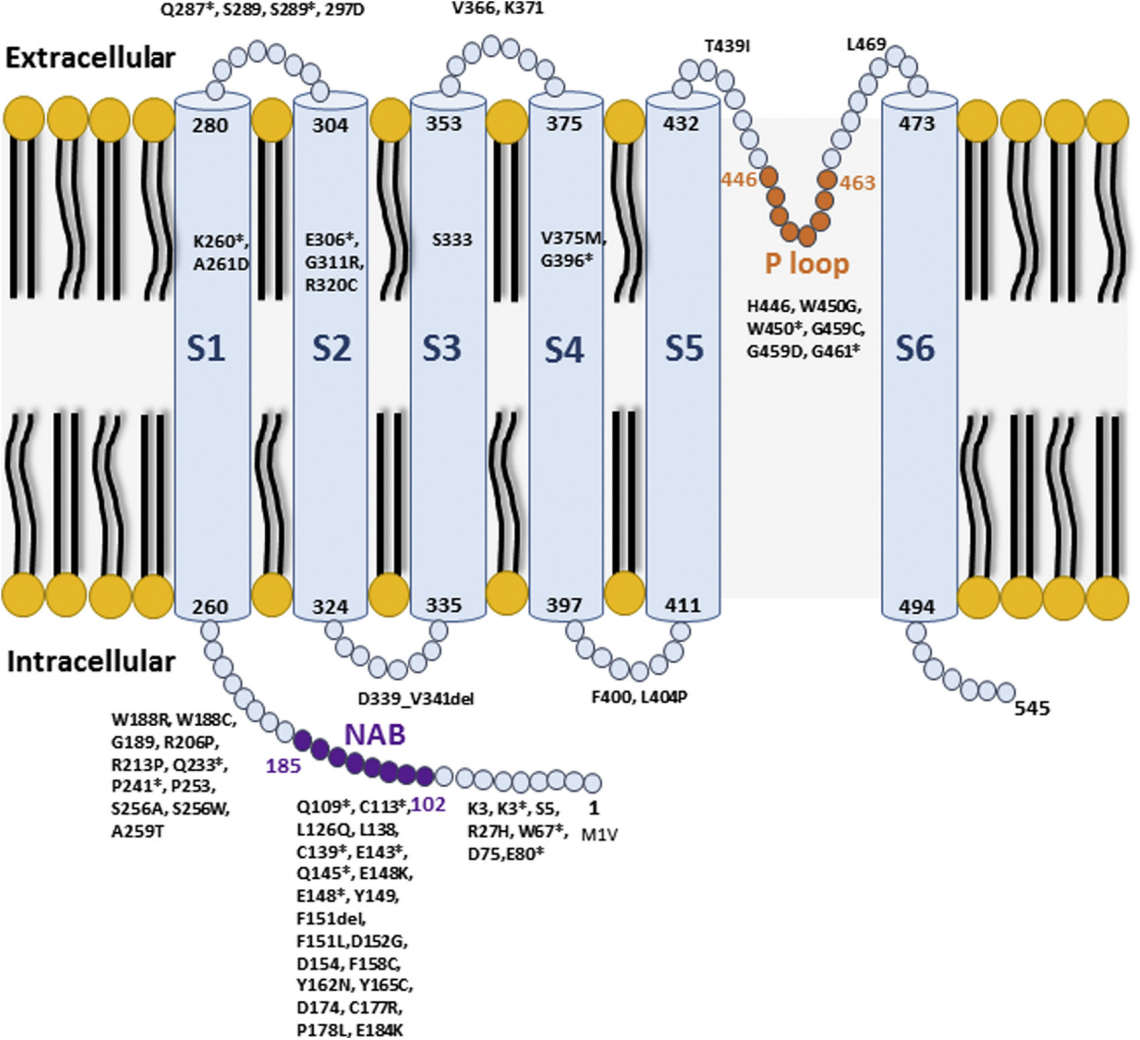
KCNV2 protein and domains. The schematic diagram shows the *KCNV2* encoded protein structure, the alpha-subunit of the potassium channel (Kv8.2), and its domains. It consists of 1) a highly conserved tetramerization domain; *N*-terminal A and B box (NAB); 2) 6 transmembrane domains (S1-S6); 3) extracellular and intracellular loop segments; and 4) an ultraconserved potassium selective motif in the pore-forming loop between S5 and S6 (P loop). The distribution of the missense variants identified are detailed in the results section.

*KCNV2*-associated retinopathy (OMIM #610356) was first described in 1983 by Gouras and associates^1^ as cone dystrophy with nyctalopia and supernormal rod responses. “Cone dystrophy with supernormal rod response” was later linked to a 1.5-Mb region on chromosome 9p24 and variants in *KCNV2* (OMIM #607604).^2^ *KCNV2* encodes a modulatory subunit (Kv8.2) of a voltage-gated potassium channel (Figure 1).^2^ In situ hybridization demonstrated *KCNV2* expression in human rod and cone photoreceptors.^2^ Abundant Kv8.2 (KCNV2) expression is also reported in the photoreceptor layer of the mouse retina.^3^ The Kv8.2 subunit interacts with different Kv2 channels in rods and cones, giving rise to potassium currents that shape the photoreceptor membrane potential.^4^

*KCNV2*-associated retinopathy represents an uncommon autosomal recessive retinal disorder and a leading cause of inherited cone-rod dystrophy.^5^ Disease-causing variants in *KCNV2* are present in a substantial fraction (2%-5%) of different cohorts with the broad clinical diagnoses of a cone dysfunction/dystrophy, suggesting that Cone dystrophy with supernormal rod response may be underdiagnosed.^6^, ^7^, ^8^ In total, we identified 114 cases described in the literature, in 22 studies and case reports.^9^ Clinically, *KCNV2* is characterized by variable age of onset, usually in infancy or early childhood, color vision defects (most commonly in the red–green axis), impaired adaptations to different light conditions, mild photophobia and nyctalopia.^10^, ^11^, ^12^, ^13^, ^14^, ^15^ In young patients, clinical presentation can be variable, the most common presentation being abnormal head position, head shaking, and nystagmus that improves with time.^10^ The photopic full-field electroretinogram (ERG) shows evidence of generalized cone system dysfunction, with scotopic ERGs revealing unusual rod system involvement, whereby responses to dim flashes are attenuated and markedly delayed, and ERG b-waves to strong flashes being relatively large, with a characteristic strong flash ERG waveform shape. The ERG abnormalities are pathognomonic, with directed molecular genetic testing confirming the diagnosis.^12^^,^^16^, ^17^, ^18^, ^19^, ^20^, ^21^, ^22^ Many of the aforementioned findings are based on single reports and small cohorts, and questions about the ERG spectrum of the disease, stability over time, and clinical presentation need further investigation, given the inherent variability in inherited retinal diseases.^5^^,^^23^

We present the first report of a multicenter international collaborative retrospective cohort investigation of 117 individual adults and children with disease-causing variants in *KCNV2*. The current report provides a detailed description of the genetics, electrophysiology, and clinical course of the disease. This information is of particular importance for improving genetic counselling and advice on prognosis and provides a crucial step toward the design of a prospective natural history study and therapeutic clinical trial for *KCNV2*-associated retinopathy. The study also identifies a cohort of molecularly confirmed patients who may be suitable candidates for treatment and further investigations of disease natural history.

## Methods

The study protocol adhered to the tenets of the Declaration of Helsinki and received approval from all local ethics committees of the participating institutions. Informed consent was obtained from all adult subjects, whereas informed consent and assent were obtained from parents and children, respectively.

### Patient Identification

Inclusion criteria for the current study were the molecular and/or phenotypic confirmation of *KCNV2*-associated retinopathy. A search was performed in the genetics database of Moorfields Eye Hospital, London, UK, the RetDis Biobank and database of the Center for Ophthalmology, University of Tübingen, Germany, and major referral centers across the globe were contacted for participation in the study.

### Molecular Diagnosis

A combination of direct Sanger sequencing and next-generation sequencing, including panels of retinal dystrophy genes, whole exome sequencing (WES) and whole genome sequencing (WGS), was used to identify variants in *KCNV2* in the different referral centers. All recruited patients were reassessed for their detected variants. Minor allele frequency for the identified variants in the general population was assessed in the Genome Aggregation Database datasets (available at: http://gnomad.broadinstitute.org/). The Combined Annotation Dependent Depletion score was calculated for all variants; a score >15 is usually considered mildly pathogenic and a score >20 is strongly indicative.^24^ The deleterious annotation of genetic variants using neural networks score was calculated for single nucleotide variants. A deleterious annotation of genetic variants using neural networks score >0.9 is considered suggestive of pathogenicity.^25^ The evolutionary conservation of the affected amino acid residues was evaluated with Clustal Omega software (available at: http://www.ebi.ac.uk/Tools/msa/clustalo/). Classification of all detected variants was also performed based on the guidelines of the American College of Medical Genetics and Genomics.^26^

### Clinical Assessment

All patients were seen by inherited retinal disease specialists at referral sites. Available clinical notes were reviewed, including best-corrected visual acuity (BCVA), refraction, fundoscopy, and slit-lamp biomicroscopy findings. BCVA analysis was performed using the average of right and left eye and included cross-sectional and longitudinal analysis. Spherical equivalent was calculated for refractive errors. Mean myopic/hyperopic spherical equivalents for both eyes, were classified as: mild ≠ 0 diopters (D) to ±3.0 D, moderate ±3.0 D to ±6.0 D, and high for ≥ ±6.0 D.

### Electrophysiologic Assessment

The quantitative ERG analysis was restricted to recordings from a single referral center (Moorfields Eye Hospital, London, UK) to avoid variability caused by different test protocols and/or the use of different types of recording electrodes. Full-field ERG and pattern ERG were performed using gold foil electrodes and incorporated the International Society for Clinical Electrophysiology of Vision standards,^27^^,^^28^ except in 4 young children who underwent ERG testing with skin electrodes using modified protocols.^29^ Photopic on-off ERGs were also recorded (stimulus duration, 200 ms) in adults.^30^ Patient data were compared with ERGs from a control group of healthy subjects (age range, 10-79 years) which included validated recordings for DA 0.01 (n = 117), DA 10.0 (n = 141), LA 3.0 30 Hz (n = 131), and the LA 3.0 (single flash cone) ERG (n = 109).

### Statistical Methods

Statistical analysis was carried out with SPSS Statistics for Windows (version 22.0; IBM Corp, Armonk, New York, USA). Significance for all statistical tests was set at *P* < .05. The Shapiro-Wilk test was used to test for normality for all variables.

## Results

### Demographics

In total, 117 patients with likely disease-causing variants in *KCNV2* were ascertained for phenotyping, from 12 tertiary referral centers in 9 countries (UK, Germany, Spain, the Netherlands, United Arab Emirates, Israel, Japan, USA, and Canada). The cohort included 59 females (50.4%) and 58 males (49.6%). Data relating to 68 patients were previously reported in part in at least 1 study (Supplemental Table 1; Supplemental Material available at AJO.com). The baseline age and the follow-up time is indicated below on each individual assessment.

### Molecular Genetics

Ninety-six families were identified. Twenty-one families contributed 2 patients each, with the remaining 75 patients being single cases. In 113 patients (96.6%), 2 *KCNV2* disease–causing variants were identified: 52 (44.4%) patients were compound heterozygotes and 61 (52.1%) patients harbored homozygous variants. For 4 patients, only 1 variant was identified (3.4%); however, since the patients were previously reported^12^^,^^16^ and had a characteristic phenotype, they were included in the analysis. Supplemental Table 1 summarizes the molecular findings of each patient.

In total, 230 disease-causing alleles were identified, corresponding to 75 different *KCNV2* variants. Supplemental Table 2 presents all the variants identified in the cohort, their minor allele frequencies in the general population (Kaviar,^31^ Genome Aggregation Database,^32^ Tommo,^33^ KRGDB,^34^ and HGVD,^35^ databases) and their predicted effect. More than one third of the alleles (35.7%) presented as 1 of 4 recurrent variants: 1) c.427G>T p.(Glu143∗) (n = 29, 12.61%); 2) c.1381G>A p.(Gly461Arg) (n = 26, 11.30%); 3) c.778A>T p.(Lys260∗) (n = 17, 7.39%); and 4) c.325C>T p.(Gln109∗) (n = 10, 4.34%). The aforementioned alterations were identified in 45 families (46.9%). The variant p.(Glu143∗) was identified in 12 families from only 2 referral centers (Moorfields Eye Hospital, London, UK and Cleveland Clinic Abu Dhabi, Abu Dhabi, United Arab Emirates), with 11 of the families being of Arabian Peninsula origin. The other 3 common variants were identified in various referral centers. Table 1 summarizes the most frequent *KCNV2* variants, and Supplemental Table 2 presents the number of alleles identified and the frequency of all the alterations identified.

**Table 1:** Most Frequent *KCNV2* Variants

Variant		Alleles, n = 230		Families, n = 96	
DNA Alternation^a^	Protein Effect	Times Identified, n	Frequency, %	Times Identified, n	Frequency, %
c.1381G>A	p.(Gly461Arg)	26	11.3	19	19.8
c.427G>T	p.(Glu143∗)	29	12.6	12	12.5
c.778A>T	p.(Lys260∗)	17	7.4	10	10.4
c.325C>T	p.(Gln109∗)	10	4.3	4	4.2
Total		82	35.7	45	46.9

a Reference sequence NM_133497.4.

Of 75 alterations identified, 28 (37.3%) were novel to the best of our knowledge (Supplemental Table 3). Most of the variants (n = 28, 37.3%), were missense, followed by nonsense (n = 18, 24%) and frame-shifting indels (n = 18, 24%). The missense variants identified in our cohort were not evenly distributed throughout the full length of the gene. Ten missense variants (35.7%) were clustered in the highly conserved tetramerization domain (*N*-terminal A and B box [NAB]) that facilitates interaction between compatible K_v_ subunits. Interestingly, 7 variants (25%) were identified in the extracellular link between the NAB and the first transmembrane domain (S1), and 1 (3.6%) in the intracellular part before the NAB. Only 5 variants (17.9%) were localized in the 6 transmembrane domains (S1-S6), and 1 variant (3.6%) in one of the intracellular loop segments. In the ultraconserved potassium selective motif in the pore-forming loop between S5-S6 (P loop), 4 variants (14.3%) were identified, including the most common missense variant (c.1381G>A p.[Gly461Arg]). Other commonly encountered alleles also clustered within highly conserved domains (eg, c.427G>T p.[Glu143∗] and c.325C>T p.[Gln109∗] within the NAB domain). Figure 1 presents a graphical representation of the gene and its domains. Supplemental Figure 1 shows the evolutionary conservation of the affected amino acid residues. Table 2 summarizes the variants by type and frequency.

**Table 2:** *KCNV2* Gene Alteration Types

Alteration Type	Alterations, n	Frequency, %
Missense	28	37.3
Nonsense	18	24.0
Frameshift	18	24.0
Exon(s) deletion	3	4.0
In-frame deletion	2	2.7
Stop codon lost	2	2.7
Whole gene deletion	2	2.7
Splicing site	1	1.3
Start codon lost	1	1.3

### Disease Onset

Age of onset was available for 95 patients, for 65 of them the age was recorded in years, and for 30 of them as “infancy” (0-2 years of age), “early childhood” (3-8 years of age), and “middle childhood” (9-11 years of age). Table 3 summarizes the age of onset by developmental stage for all 95 patients. The mean (±SD, median) age of onset for the patients with available age (n = 65) was 3.9 years old (SD ± 3.0; median 3 years). Twelve patients (18.5%) were symptomatic at birth. All patients were symptomatic before 12 years of age (range, 0-11 years). Age at baseline examination is detailed in the BCVA section below.

**Table 3:** Clinical Findings

Age of Disease Onset, n = 95	Frequency, n (%)
Infancy (birth to 2 years old)	30 (31.6)
Early childhood (3-8 years old)	45 (47.4)
Middle childhood (9-11 years old)	25 (21.1)
Common symptoms and findings, n = 101	
Reduced BCVA	101 (100.0)
Reduced color vision^a^	55 (78.6)
Photophobia	54 (53.5)
Nyctalopia	44 (43.6)
Nystagmus	39 (38.6)
Clinical presentation, n = 101: Reduced BCVA and:	
Only reduced BCVA	17 (16.8)
Nyctalopia, photophobia, and reduced color vision	12 (11.9)
Reduced color vision and photophobia	10 (9.9)
Nystagmus, photophobia, and reduced color vision	9 (8.9)
Nystagmus, nyctalopia, photophobia, and reduced color vision	7 (6.9)
Reduced color vision	7 (6.9)
Nyctalopia and photophobia	7 (6.9)
Nystagmus and nyctalopia	5 (5.0)
Nystagmus and reduced color vision	5 (5.0)
Nystagmus	4 (4.0)
Nyctalopia	4 (4.0)
Nystagmus and photophobia	4 (4.0)
Nyctalopia and reduced color vision	3 (3.0)
Nystagmus, nyctalopia, and photophobia	3 (3.0)
Nystagmus and nyctalopia, and reduced color vision	2 (2.0)
Photophobia	2 (2.0)
Refraction: Spherical equivalent, n = 60	
Myopic	
Mild (0 D to −3.0 D)	11 (18.3)
Moderate (−3.0 D to −6.0 D)	14 (23.3)
High (≥−6.0 D)	19 (31.7)
Hyperopic	
Mild (0 D to +3.0 D)	14 (23.3)
Moderate (+3.0 D to +6.0 D)	1 (1.7)
0 D spherical equivalent	1 (1.7)

BCVA = best-corrected visual acuity; D = diopter.

a Only 70 of the 101 patients had specific documented color vision test.

### Symptoms and Clinical Examination Findings

One hundred and one patients had recorded symptoms at disease onset. A universal finding was decreased visual acuity (100%). The clinical presentation varied (Table 3), but common symptoms included reduced color vision (78.6%), photophobia (53.5%), and nyctalopia (43.6%), with nystagmus occurring in a large proportion of patients (38.6%). Twenty-nine patients (28.7%) had photophobia and nyctalopia, with or without other symptoms. Patients with nystagmus tended to be younger, and the nystagmus was observed to reduce with increasing age. Beyond the 5 common signs, 5 young children had strabismus, and 3 infants had head shaking.

On clinical examination, all patients had clear ocular media. On fundoscopy the peripheral retina was normal in most, including 4 with a tigroid appearance, but 4 patients showed subtle pigment mottling. Macular appearance was variable, being normal in a minority, but in most cases, it ranged from an absent or reduced reflex to a bull's eye appearance, and in advanced cases retinal pigment epithelium hyperpigmentation and atrophic changes were common.

### Visual Acuity

**Figure 2:**
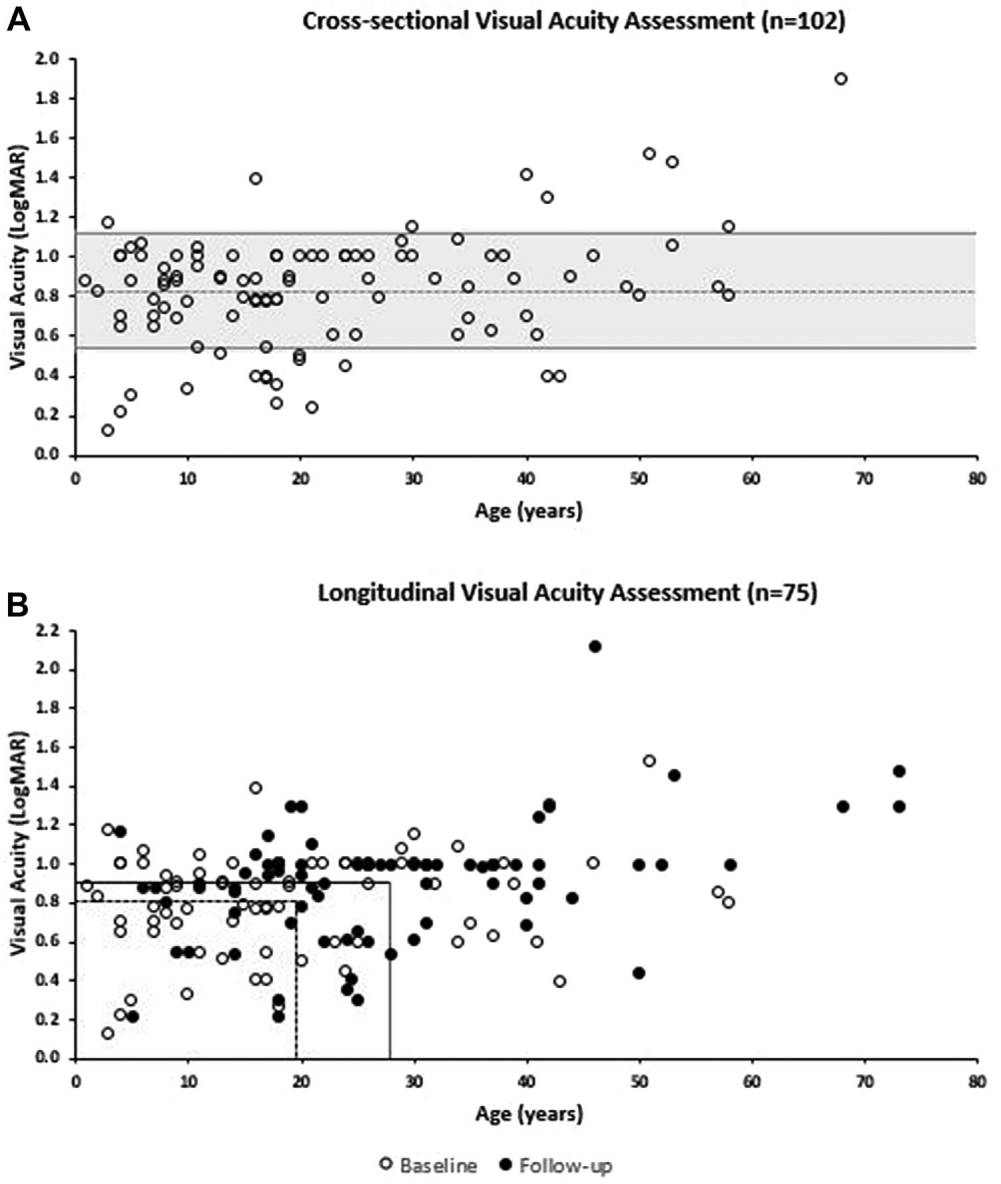
Best-corrected visual acuity (BCVA) assessment. A. Cross-sectional assessment of BCVA based on data from 101 patients. The dashed line marks the mean of the cohort (0.83 logarithm of minimal angle of resolution [logMAR]). More than two-thirds of the patients (68.2%) had a BCVA between 0.54 and 1.12 logMAR (shadowed area between lines marking ± SD). B. Longitudinal assessment of BCVA on data from 75 patients. The dashed lines mark the mean age and the mean BCVA at baseline—19.5 years and 0.81 logMAR, respectively. The continuous lines mark the mean age and mean BCVA at follow-up—27.9 years and 0.90 logMAR, respectively. After a mean follow-up of 8.4 years, the mean BCVA (±SD) decreased by 0.09 logMAR. The annual rate for the cohort was 0.011 logMAR per year.

BCVA was assessed cross-sectionally and longitudinally. One hundred and two patients had BCVA available at ≥1 visits. None of the patients had any other vision-limiting disease. The mean age (±SD, median, range) of the group was 22.1 years (±15.5, 18, 3-68 years), and their mean BCVA (±SD, median, range) was 0.83 logMAR (±0.29, 0.88, 0.22-1.9 logMAR) at baseline. Figure 2, A presents all the available cross-sectional data. There was a weak statistically significant correlation between the mean BCVA for right and left eye, and the baseline age (*r* = 0.20, *P* = .04, Spearman correlation coefficient). The weak correlation can reflect the severe decrease in BCVA early in life and a further slow decline with age.

Seventy-five patients had available longitudinal data. The mean age (±SD) at baseline and the follow-up visit was 19.5 years (±13.5 years) and 27.9 (±15.2 years), respectively. After a mean follow-up of 8.4 years (±SD [range], ±7.3 [1-31] years), the mean BCVA (±SD) decreased from 0.81 ± 0.27 logMAR to 0.90 ± 0.31 logMAR). Figure 2, B presents the longitudinal BCVA data. Despite the overlap of the scatter baseline and follow-up data, the follow-up BCVA was significantly worse (*P* = .005, *z* = 2.78, Wilcoxon signed rank matched-pairs test).

### Refraction

Refraction data were available for 60 patients, with all of them being phakic, and all except 1 having a refractive error. The mean age of refraction (±SD, range, median) was 21.6 years (±14.6, 3-58, 17.5 years). The mean spherical equivalent was −3.57 D (range, −14.75 D to +2.75 D) in the right eye and −3.60 D (range, −16.25 D to +3.75 D) in the left eye. The median spherical equivalent was −3.75 D and −3.19 D for the right and left eyes, respectively. Table 3 categorizes mean spherical equivalent of both eyes for all patients. High myopia (≥−6.0 D) was a common finding (31.7%).

### Electrophysiologic Assessment

**Figure 3:**
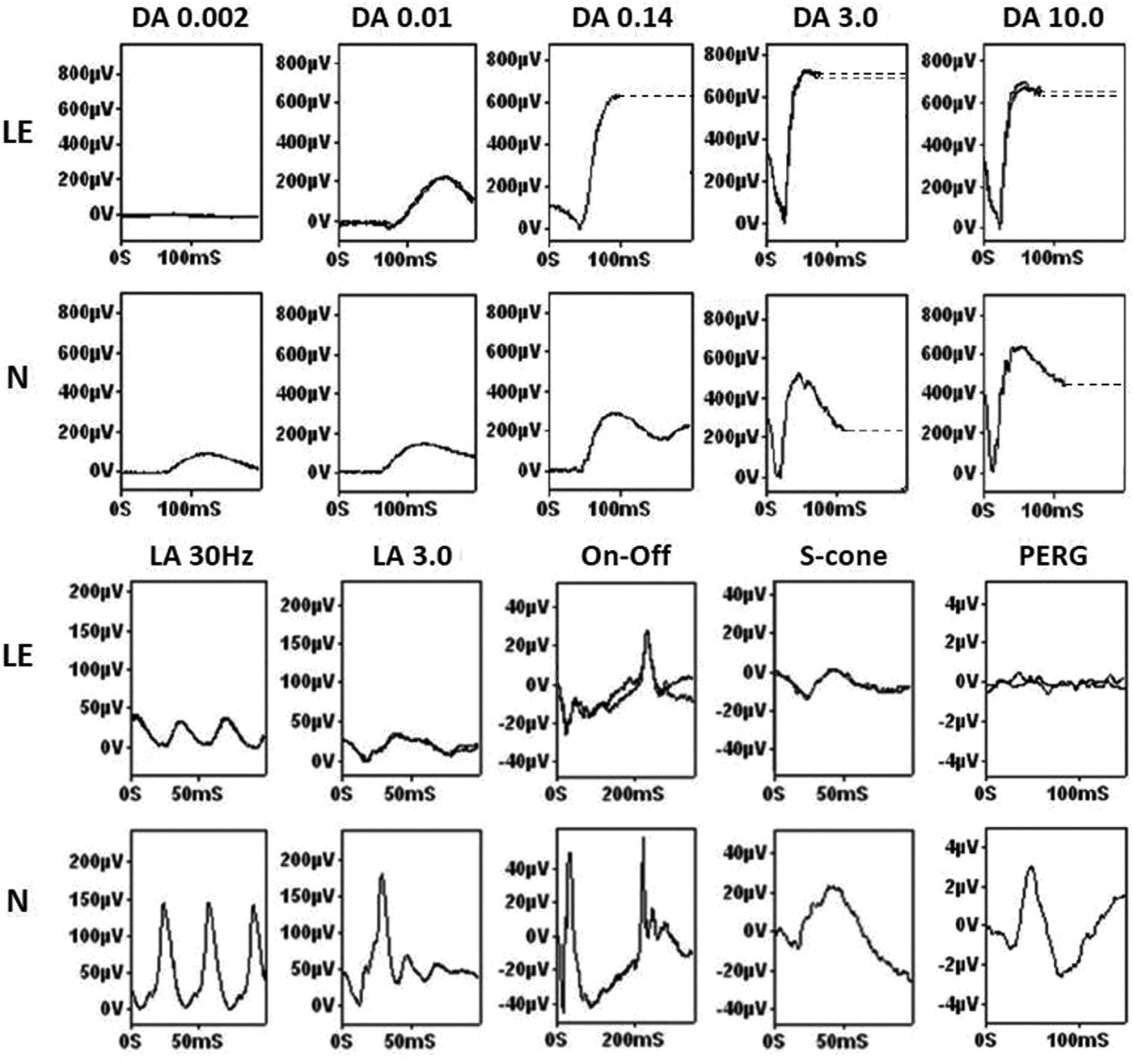
Full-field electroretinography (ERG) and pattern ERG recordings in a case of *KCNV2*-associated retinopathy. The dark-adapted (DA) responses (top panels) show the pathognomonic features. To the dimmest flash the (DA 0.002) ERG was undetectable and the DA0.01 ERG delayed and subnormal. As flash strength increased up to 3 cd.s.m^−2^, there was abnormal increased enlargement of the ERG. The DA 10 (strong flash) ERG a-wave trough had a characteristic broad shape with a late negative component and the b-wave was disproportionately large relative to the attenuated dim flash responses. Light-adapted (LA) 30-Hz flicker (LA 30 Hz) and single flash cone (LA3) ERGs were delayed and subnormal (bottom panels). The photopic on-off ERG (stimulus duration, 200 ms) showed a delayed and markedly reduced b-wave (an electronegative on-response) and delay and mild reduction of the d-wave (the off-response). The S-cone ERG was simplified and reduced. The pattern ERG P50 component was undetectable in keeping with severe macular dysfunction, typical of the disorder. Representative control recordings from an unaffected individual are shown for comparison (N). All patient recordings showed a high degree of interocular symmetry, are shown from 1 eye only, and are superimposed to demonstrate reproducibility, with the exception of the DA 0.14 ERG (single recording). Broken lines replace eye movement artefacts seen after the b-waves for clarity. LE = left eye, N = normal control, PERG = pattern ERG.

International Society for Clinical Electrophysiology of Vision dark-adapted (DA) ERGs showed a pathognomonic combination of features in all subjects tested (n = 45; age range, 4-59 years), and all had abnormal light-adapted (LA) ERGs. Figure 3 presents a typical example and description of the features compared with a normal control subject. Median peak times and amplitudes for standard ERG components (DA 0.01 ERG; DA 10 ERG a and b-waves; LA 30 Hz ERG, and LA 3 ERG a- and b-waves) were significantly different compared with the control group (Table 4). The mean peak time differences (delays) for the DA 0.01 ERG and DA 10 ERG a- and b-waves were 61 ms, 12 ms, and 6 ms, respectively, and the mean delay in LA 30 Hz ERG peak time was 9 ms. The mean amplitude reductions for the DA 0.01 ERG, DA 10 ERG a-waves, LA 30 Hz, and LA 3 ERG a- and b-waves were 55%, 21%, 48%, 47%, and 74%, respectively; the DA 10 ERG b-waves showed a mean increase of 18% compared with the mean for the control group, but there was marked variation (range, −24% to +70%).

**Table 4:** The Amplitudes and Peak Times (Median and Percentiles) of the Main Full-Field Electroretinography Components in a Control Group Compared with *KCNV2* Retinopathy

Stimulus	Component	Parameter	Control Group			*KCNV2*			*P* Value
			5th	Median	95th	5th	Median	95th	
DA 0.01	b-wave	Amplitude	110	210	370	51	114	350	1.58 × 10^−8^^a^
		Peak time	77	98	113	130	153	182	3.76 × 10^−13^^a^
DA 10	a-wave	Amplitude	185	319	435	166	271	365	2.32 × 10^−8^^a^
		Peak time	9	11	13	18	23	25	2.73 × 10^−14^^a^
	b-wave	Amplitude	290	465	645	380	575	797	5.48 × 10^−3^^a^
		Peak time	41	49	55	45	54	62	1.32 × 10^−6^^a^
LA 30-Hz	Peak	Amplitude	55	105	180	21	40	57	2.16 × 10^−12^^a^
		Peak time	23	25	30	30	34	38	1.09 × 10^−14^^a^
LA 3	a-wave	Amplitude	20	35	65	12	23	40	8.72 × 10^−11^^a^
	b-wave	Amplitude	90	155	250	25	50	85	1.10 × 10^−13^^a^

DA = dark adapted; LA = light adapted.

Amplitudes are in microvolts and peak times are in milliseconds. Statistical significance was established using the Mann-Whitney *U* test.

a Statistically significant following Bonferroni correction.

**Figure 4:**
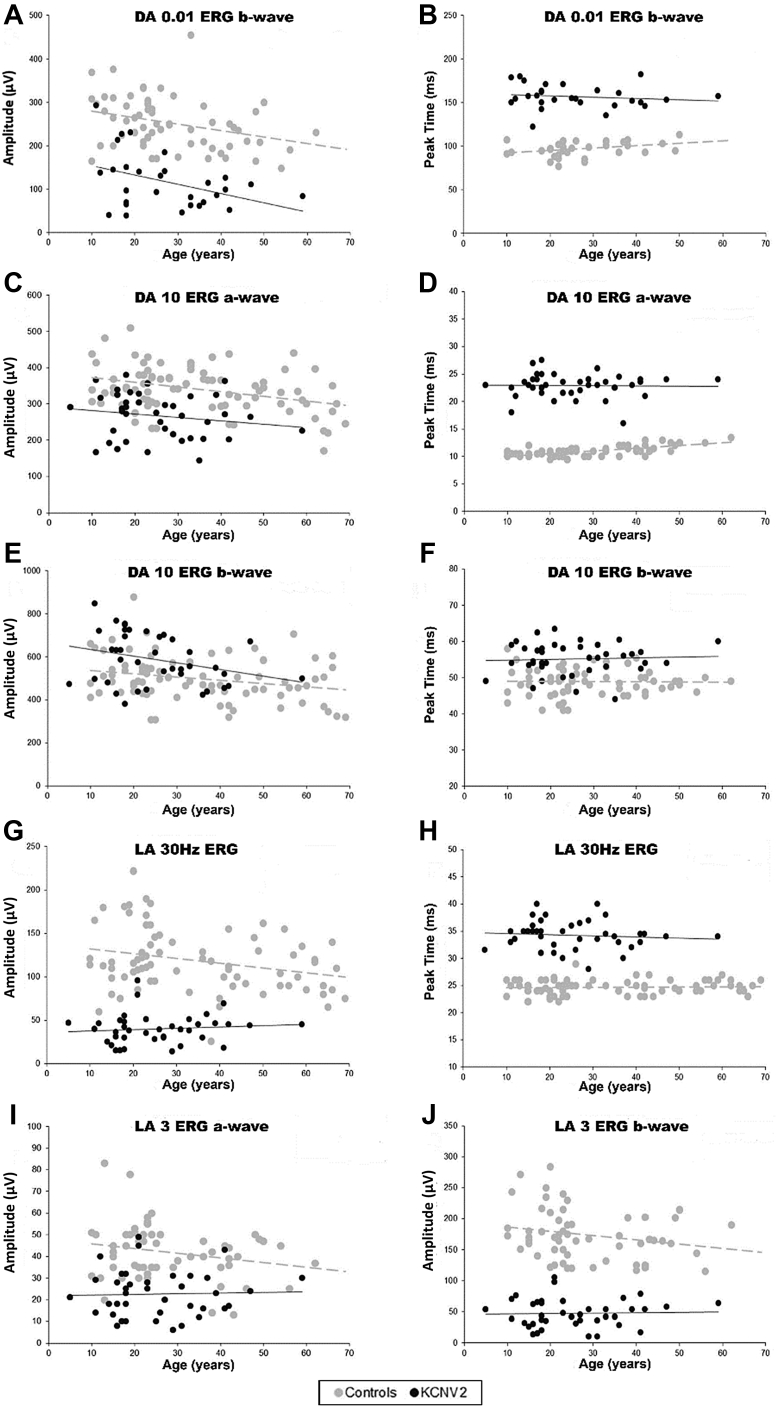
Scatter plots for electrophysiologic parameters and age. The major full-field electroretinography (ERG) component peak times and amplitudes in *KCNV2* patients (filled circles) and unaffected control subjects (gray circles) are plotted against age. Data are shown for the dark-adapted (DA) 0.01 ERG (A and B), DA10.0 ERG a- and b-waves (C through F), light-adapted (LA) 30-Hz ERG (G and H), and for the amplitude of the LA 3 ERG a- and b-waves (I and J). Regression lines are shown for the *KCNV2*-associated retinopathy (solid line) and control (gray broken line) data. See text for details.

The DA 0.01 ERG and DA 10 ERG a- and b-wave amplitudes tended to be larger in younger patients with *KCNV2*, but there was wide variability and the mean rate of age-related decline was indistinguishable from that seen in the control group (Figure 4, A, C, and E). The DA ERG peak times were similar at all ages (Figure 4, B, D, and F). The LA 30 Hz and LA 3 ERG amplitudes and peak times showed no evidence of worsening with increasing age (Figure 4, G through J). PERGs were undetectable in all patients tested.

Of the 4 young children tested using periorbital skin electrodes, noisy ERG recordings in the youngest (aged 4 years) were equivocal; in the others (aged 5, 5, and 10 years) the ERGs showed features consistent with *KCNV2*-associated retinopathy (Supplemental Figure 2).

## Discussion

This international multicenter investigation explores the clinical phenotype and aspects of the natural history of *KCNV2*-associated retinopathy, in a large cohort of molecularly proven patients, over a wide range of ages. The findings of the current report (number 1) confirm the early onset of disease associated with severe visual impairment and pathognomonic ERG features, and suggest a high degree of functional stability over time. The study establishes a cohort of candidates that could potentially benefit from the development of novel therapeutics, such as gene replacement therapy, gene editing, and stem cell–based therapies.

### Genetics and Disease Epidemiology

The genetic background of cone dystrophy with supernormal rod response is uniform and strictly associated with biallelic variants in *KCNV2*. Early genetic investigations—before the mapping of the disease locus—speculated upon an association with *PDE6H* variants.^36^ A later study (and the current report) supported a unique genotype–phenotype correlation related to *KCNV2* variants,^13^ and *PDE6H* variants are now considered a rare cause of achromatopsia.^37^^,^^38^ All the patients in the current study had pathognomonic ERG findings and molecular confirmation of variants in *KCNV2*. The 4 patients with only 1 heterozygous variant identified were previously reported, and no further more sensitive methods for identification of the second variant have been performed so far.^12^^,^^16^ Robson and associates^12^ published a cohort of 24 patients with 18 of them molecularly confirmed; subsequently, another 5 had genetic testing, with all yielding biallelic *KCNV2* variants (Supplemental Table 1). The current study reported a further 28 novel *KCNV2* variants—a significant addition to the 95 previously reported variants.^9^

With the recognition of the pathognomonic ERG phenotype, targeted *KCNV2* screening is of high yield as previously suggested.^17^ In the same study, *KCNV2*-associated retinopathy was identified as the second most common cause (11.3%) of paediatric inherited retinal disease in an Emirati cohort of 71 patients.^17^ The increased prevalence of the disease in the aforementioned study may be a result of a founder effect.^17^ The variant c.427G>T p.(Glu143∗) was the second most common in the cohort, with 11 of the 12 families identified being of Arabian Peninsula origin. The variant was also the most common variant in a previously reported Saudi cohort.^39^ *KCNV2*-associated retinopathy accounts for 0.7% and 0.25% of families with molecularly confirmed inherited retinal disease in the United Kingdom and Germany, respectively.^40^^,^^41^ In the United States, disease frequency was calculated to 1 in 850,000 individuals and an estimated yield of 5 new cases per year.^42^ We identified 4 variants (Table 1) which are involved in half of the affected families. The *KCNV2* study group highlights the worldwide distribution of the disease without being able to account for its prevalence. *RPE65*-associated Leber congenital amaurosis, the disease with the first adeno-associated virus–based gene therapy treatment approved by the US Food and Drug Administration a genotype investigated in detail, has an estimated frequency of 1 in 576,667 individuals, with an estimated 7 new cases annually in the United States.^42^ *KCNV2* is a 2-exon gene encoding for 545 amino acids^9^ that can thus also be accommodated in adeno-associated virus vectors, making it an attractive target for gene therapy.

### Clinical Presentation and Disease Course

*KCNV2*-associated retinopathy is a severe early onset disease. We identified *KCNV2*-associated retinopathy as a childhood onset disease, with more than half of the patients being symptomatic before 3 years of age. One in 10 patients was symptomatic at birth, with nonspecific signs, such as nystagmus and head shaking, which pose a diagnostic challenge given the similarity to cone dysfunction syndromes, including achromatopsia, and forms of Leber congenital amaurosis,^43^^,^^44^ or even nonretinal conditions, such as spasmus nutans, when the fundus appearance is grossly normal,^39^ illustrating the importance of ERG testing and genetic screening for definitive diagnosis. Despite the lack of quantification, high-frequency pendular nystagmus tends to decrease over the span of several years, as previously reported.^39^^,^^45^ The combination of photophobia and night vision difficulties was present in one third of our cohort and is an important diagnostic clue that can help differentiate the disease from other cone dysfunction syndromes and early stage cone-rod dystrophies (not associated with night blindness). Light sensitivity and nyctalopia may be targeted for the development of relevant potential endpoints for a future clinical trial. Photoaversion can be quantified,^46^^,^^47^ and nyctalopia can be tested with mobility assessments at different light levels.^48^

BCVA was severely reduced in all patients from an early age (Figure 2, A) and slowly worsened with age (Figure 2, B). Halt or slowing of BCVA loss may not be ideal as a primary endpoint for clinical trials of short duration,^49^ given that the mean annual change of 0.01 logMAR corresponds to 1 Early Treatment Diabetic Retinopathy Study letter every 2 years. Also, the severely reduced BCVA from early childhood (Figure 2), the early onset of disease, and previously documented evidence of macular atrophy with increasing age^12^ underline the need for early intervention. An interesting finding is the universal presentation of refractive error; no specific error was associated with the disease (Table 3), although >30% of cases had high myopia (≥−6.0 D). The clinical characteristics have been previously described in smaller cohorts of 1 to 24 patients.^8^^,^^13^^,^^15^^,^^16^^,^^20^^,^^21^^,^^39^^,^^45^^,^^50^^,^^51^ We were able to establish the frequency of symptoms and elaborate on the age of onset and BCVA natural history in a much larger number of patients.

### Electrophysiology

Full-field ERG findings are specific for the disease (Figure 3) despite variability in the absolute peak times and amplitudes of the main ERG components, as detailed in this study (Figure 4). In *KCNV2*-associated retinopathy, the scotopic dim flash (DA 0.01) ERG is delayed in all cases and is subnormal in the majority. The strong flash (DA10.0) ERG a-wave may be of mildly subnormal to normal amplitude and the b-wave is relatively large compared with dim flash responses; the b-wave amplitude often falls within the normal range, with a minority being abnormally large (“supernormal”). Comparison of the main ERG components with age over 6 decades shows a mean age-associated decline in amplitude at a rate similar to that in the unaffected control group (Figure 4). This is consistent with relatively stable peripheral retinal dysfunction and is in keeping with previous published evidence of nonprogressive peripheral retinal dysfunction over 15 years, despite worsening macular atrophy.^50^

All patients in the current cohort had detectable but abnormal LA ERGs indicative of generalized cone system dysfunction, including a child tested with skin electrodes (Supplemental Figure 1). A previous report of skin ERGs in a child highlighted the possibility of unusually severe cone system dysfunction in association with homozygous deletion of *KCNV2*.^52^ In contrast, Zobor and associates^19^ did not observe a genotype–functional phenotype correlation in patients with no protein expression (n = 3) or residual protein expression. Electrophysiologic data from a cohort of 10 patients were reported as being consistent with a postphototransduction but pre–inner nuclear layer dysfunction.^11^ Another study (n = 6) suggest altered function of the inner retina, based on the reduced oscillatory potentials.^19^ Stockman and associates^53^ psychophysically characterized the disease and suggested an intact phototransduction process.

### Limitations and Future Directions

Our study has many strengths, including the size of the cohort, which is the largest to date evaluating *KCNV2*-associated retinopathy, the age range of patients, and that of the ERG control group, allowing age-associated evaluation of retinal function. In addition to clinical diagnosis and/or ERG, all included patients were molecularly confirmed with pathogenic variants in *KCNV2*. The study includes patients from referral centers with wide geographic distribution and is therefore less susceptible to selection bias of study population and proving the benefits of international collaboration in rare diseases. Inherent limitations to the study relate to its retrospective nature; not all data were available for all patients and many of the available data were acquired by different protocols and methods (eg, ERG and genetic testing). The aforementioned limitations were ameliorated per protocol analysis of the collected data. Where no reporting protocol could be used retrospectively for certain aspects of the clinical examination (eg, fundoscopy), the data were presented descriptively. ERG amplitude may be reduced and peak time may be increased, with increasing myopia, and our cohort had a negative mean spherical equivalent. A case-control approach, with matching age and spherical equivalent, may be of value for comparing the responses of the patients to the responses of normal control subjects in future prospective studies.

Report 1 aimed to present the genetics, electrophysiology, and clinical presentation. The genetic data in the current study provided a patient population that can be considered for future gene augmentation trials, insight into the disease genetic background and novel variants. Electrophysiology assessment further established the pathognomonic ERG phenotype in the context of phenotypic and age-associated variability, consistent with relatively stable peripheral retinal disease and suggesting a wide therapeutic window. The clinical presentation described further facilitates clinical diagnosis and highlights disease severity. The detailed investigation of the retinal phenotype and of structural meaningful endpoints for future trials were beyond the scope of this first report. A wide range of fundus autofluorescence abnormalities, including ring-like or bull's eye changes, central atrophy, or increased foveal autofluorescence have all been reported in the disease,^11^^,^^12^^,^^45^^,^^51^ and optical coherence tomography can show a variable degree of changes in the outer retina, ranging from ellipsoid zone disruption to diffuse outer retinal atrophy.^10^^,^^15^^,^^22^^,^^45^^,^^54^^,^^55^ In the *KCNV2* knockout mouse, approximately 80% of cones are still intact by 6 months of age compared with wild-type mice, which if similar to humans may allow for relatively late photoreceptor-directed treatment.^56^ However, further clinical and preclinical research, including prospective natural history studies, are needed to establish the optimal window for intervention, appropriate structural and functional (both retinal and visual) endpoints to monitor both safety and efficacy, and identify participants most likely to benefit.^9^ Report 2 will investigate longitudinal retinal imaging and endpoints.

## Conclusions

The current study is the first in-depth analysis and long-term longitudinal study of *KCNV2*-associated retinopathy. Despite its retrospective nature, we recruited more patients than the total number of patients published in the literature, empowering our study to provide novel insights into disease natural history. This investigation (report 1) highlighted the early onset of the disease, the severity of the clinical phenotype, the genetic background, the ERG stability, and established a cohort of patients with a wide geographic distribution, indicating the unmet need for trials of novel therapeutics.

## Supplemental Data

Supplemental Figure 2 Full-field electroretinography (ERG) and pattern ERG (PERG) recordings in a paediatric case of *KCNV2*-associated retinopathy. Full-field ERG and PERG recordings obtained with lower eyelid skin recordings from the left eye of a 5-year-old, showing diagnostic ERG waveforms. Recordings showed a high degree of interocular symmetry. Representative control recordings from an unaffected child are shown for comparison (N). The PERG was recorded before mydriasis and was undetectable, in keeping with severe macular dysfunction. Note that in this case there is a 20-ms prestimulus delay in the DA and LA single flash full-field ERGs. Patient traces are superimposed to demonstrate reproducibility. DA = dark adapted; LA = light adapted; LE = left eye; N = normal control; LE =left eye.

Supplementary Table 3

Supplemental Figure 1 Multiple alignment of 10 species of KCNV2. Evolutionary conservation of the affected amino acid residues in *KCNV2* was evaluated with Clustal Omega software (http://www.ebi.ac.uk/Tools/msa/clustalo/) and the amino acid sequence alignment was numbered in accordance with the *Homo sapiens KCNV2* sequence (ENSP00000371514). An asterisk indicates complete conservation across the 10 species. The positions of variant residues are shown, and a highly conserved tetramerization domain (*N*-terminal A and B box [NAB]) and an ultraconserved potassium selective motif in the pore-forming loop between S5-S6 (P loop) are highlighted with boxes.
