# Supplementary figures and images for: KCNV2-Associated Retinopathy: Genetics, Electrophysiology, and Clinical Course—KCNV2 Study Group Report 1
Source: Am J Ophthalmol. 2021 May;225:95–107. doi: 10.1016/j.ajo.2020.11.022 (PMC8186730; doi:10.1016/j.ajo.2020.11.022)

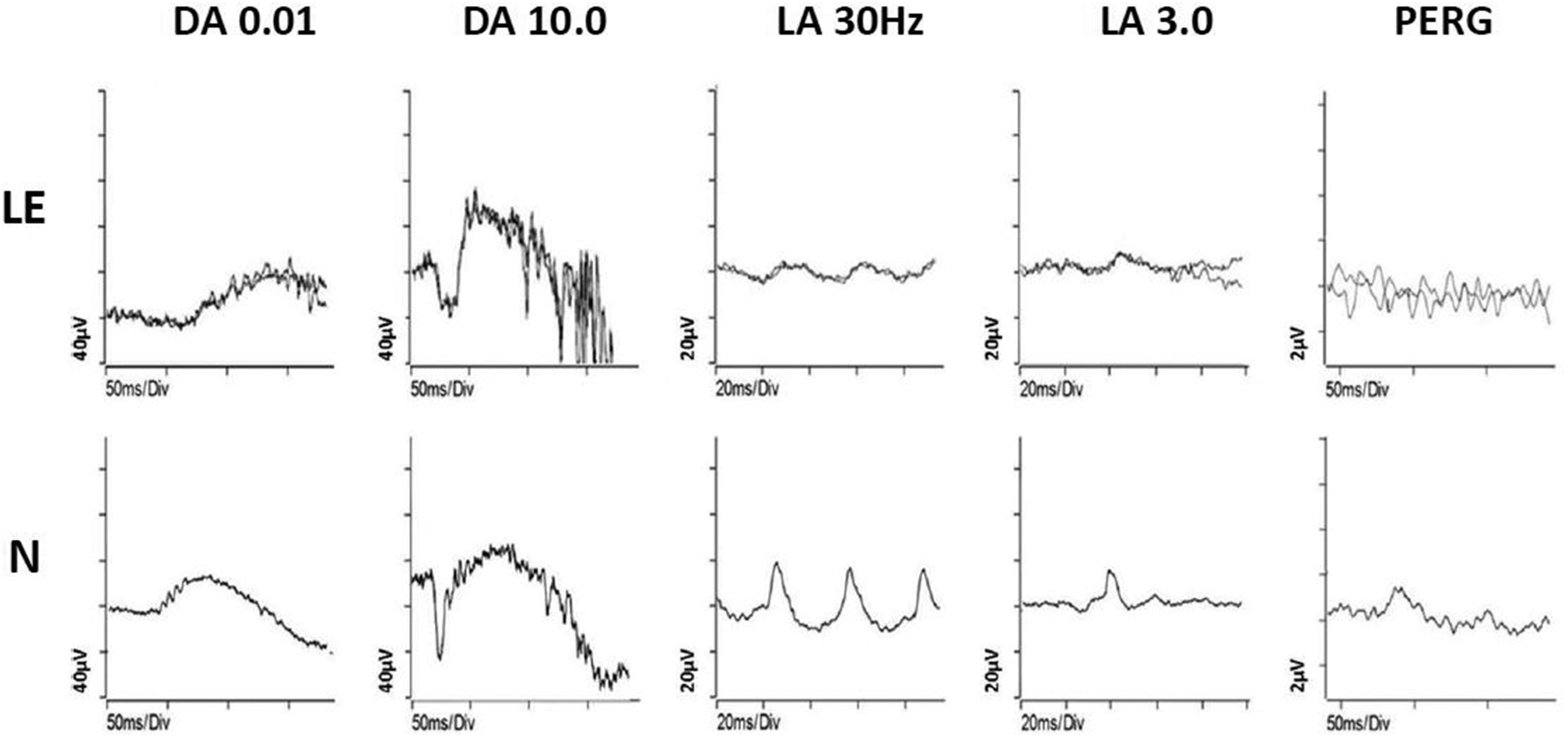

Supplement: Supplemental Figure 2 — Full-field electroretinography (ERG) and pattern ERG (PERG) recordings in a paediatric case of KCNV2-associated retinopathy. Full-field ERG and PERG recordings obtained with lower eyelid skin recordings from the left eye of a 5-year-old, showing diagnostic ERG waveforms. Recordings showed a high degree of interocular symmetry. Representative control recordings from an unaffected child are shown for comparison (N). The PERG was recorded before mydriasis and was undetectable, in keeping with severe macular dysfunction. Note that in this case there is a 20-ms prestimulus delay in the DA and LA single flash full-field ERGs. Patient traces are superimposed to demonstrate reproducibility. DA = dark adapted; LA = light adapted; LE = left eye; N = normal control; LE =left eye. [file figs1.jpg]
