# Supplementary material for: KCNV2-Associated Retinopathy: Genetics, Electrophysiology, and Clinical Course—KCNV2 Study Group Report 1
Source: Am J Ophthalmol. 2021 May;225:95–107. doi: 10.1016/j.ajo.2020.11.022 (PMC8186730; doi:10.1016/j.ajo.2020.11.022)
Supplement: Supplementary Table 1 [file mmc1.pdf]

Supplementary Table 1: *KCNV2* Variants Identified in Study-117

Pedigree ID	Patient ID	Country	Sex (M/F)	cDNA†	Protein	cDNA†	Protein	Previously published	PMID:
GC15870	MEH001	UK	M	c.916G>T	p.(Gly306*)	c.916G>T	p.(Gly306*)	Robson et al. 2010	31725702
GC16772	MEH002	UK	M	c.325C>T	p.(Gln109*)	c.325C>T	p.(Gln109*)	Robson et al. 2010	31725702
GC20749	MEH003	UK	M	c.427G>T	p.(Glu143*)	c.427G>T	p.(Glu143*)		
GC18207	MEH004	UK	F	c.1381G>T	p.(Gly461*)	c.1638G>T	p.(*546Tyr Ext61AA)	Robson et al. 2010	31725702
GC21888	MEH005	UK	M	c.1381G>A	p.(Gly461Arg)	c.494A>G	p.(Tyr165Cys)		
GC19701	MEH006	UK	F	c.1A>G	p.(Met1?)	c.766T>G	p.(Ser256Ala)		
GC18957	MEH007	UK	F	c.562T>A	p.(Trp188Arg)	c.8 11delAACA	p.(Lys3Argfs*96)		
GC16796	MEH008	UK	F	c.427G>T	p.(Glu143*)	c.767C>G	p.(Ser256Trp)	Robson et al. 2010	31725702
GC16796	MEH009	UK	M	c.427G>T	p.(Glu143*)	c.767C>G	p.(Ser256Trp)	Robson et al. 2010	31725702
GC21105	MEH010	UK	F	c.445 446delTA	p.(Tyr149Leufs*222)	c.445 446delTA	p.(Tyr149Leufs*222)		
GC16962	MEH011	UK	F	c.433C>T	p.(Gln145*)	c.776C>T	p.(Ala259Val)	Robson et al. 2010	31725702
GC4052	MEH012	UK	F	c.1199delT	p.(Phe400Serfs*54)	c.417C>A	p.(Cys139*)	Robson et al. 2010	31725702
GC16982	MEH013	UK	M	c.7A>T	p.(Lys3*)	c.7A>T	p.(Lys3*)	Robson et al. 2010	31725702
GC18268	MEH014	UK	M	c.566delG	p.(Gly189Alafs*22)	c.566delG	p.(Gly189Alafs*22)	Robson et al. 2010	31725702
GC18136	MEH015	UK	F	c.1381G>A	p.(Gly461Arg)	c.1381G>A	p.(Gly461Arg)	Robson et al. 2010	31725702
GC21109	MEH016	UK	M	c.1199delT	p.(Phe400Serfs*53)	c.417C>A	p.(Cys139*)		
GC20527	MEH017	UK	F	c.224 230delACCAGCA	p.(Asp75Glyfs*23)	c.224 230delACCAGCA	p.(Asp75Glyfs*23)		
GC20307	MEH018	UK	M	c.778A>T	p.(Lys260*)	c.1381G>A	p.(Gly461Arg)		
GC16772	MEH019	UK	F	c.325C>T	p.(Gln109*)	c.325C>T	p.(Gln109*)	Robson et al. 2010	31725702
GC20749	MEH020	UK	M	c.427G>T	p.(Glu143*)	c.427G>T	p.(Glu143*)		
GC18268	MEH021	UK	M	c.566delG	p.(G188fs*21)	c.566delG	p.(G188fs*21)	Robson et al. 2010	31725702
GC17589	MEH022	UK	F	c.1404delC	p.(His468fs*35)	c.1404delC	p.(His468fs*503)	Robson et al. 2010	31725702
GC26096	MEH023	UK	M	c.1381G>A	p.(Gly461Arg)	c.874 889dupGGCGAGGGCGGCCCAG	p.(Asp297Glyfs*80)		
GC17860	MEH024	UK	F	c.1357-? 1638+?del	p.?	c.1357-? 1638+?del	p.?	Robson et al. 2010	31725702
MEH-PP25	MEH025	UK	M	c.754A>T	p.(Lys252*)	c.775G>A	p.(Ala259Thr)		
GC25683	MEH026	UK	M	c.778A>T	p.(Lys260*)	c.778A>T	p.(Lys260*)		
GC26111	MEH027	UK	M	c.866delC	p.(Ser289Trpfs*33)	c.778A>T	p.(Lys260*)		
GC26620	MEH028	UK	M	c.1381G>A	p.(Gly461Arg)	c.484T>A	p.(Tyr162Asn)		
GC15839	MEH029	UK	M	c.377T>A	p.(Leu126Gln)	NA	NA	Robson et al. 2010	31725702
GC16701	MEH030	UK	M	c.451T>C	p.(Phe151Val)	c.1381G>A	p.(Gly461Arg)		
GC17841	MEH031	UK	F	c.451T>C	p.(Phe151Val)	NA	NA	Robson et al. 2010	31725702
GC17841	MEH032	UK	M	c.451T>C	p.(Phe151Val)	NA	NA	Robson et al. 2010	31725702
GC26268	MEH033	UK	M	c.1381G>T	p.(Gly461*)	c.442G>T	p.(Glu148*)		
GC26805	MEH034	UK	F	NC 000009.11:g.2670960 2783870del	Whole Gene Deletion	NC 000009.11:g.2670960 2783870del	Whole Gene Deletion		
GC22870	MEH035	UK	F	c.1110 1128del	p.(Lys371Alafs*77)	c.1110 1128del	p.(Lys371Alafs*77)		
GC25376	MEH036	UK	F	c.325C>T	p.(Gln109*)	c.325C>T	p.(Gln109*)		
MEH-PP37	MEH037	UK	F	c.778 A>T	p.(Lys260*)	c.778 A>T	p.(Lys260*)		
GC4128	MEH038	UK	F	c.1376G>A	p.(Gly459Asp)	c.1376G>A	p.(Gly459Asp)	Robson et al. 2010	31725702
MEH-PP3940	MEH039	UK	F	c.427G>T	p.(Glu143*)	c.427G>T	p.(Glu143*)	Robson et al. 2010	31725702
MEH-PP3940	MEH040	UK	F	c.427G>T	p.(Glu143*)	c.427G>T	p.(Glu143*)	Robson et al. 2010	31725702
MEH-PP41	MEH041	UK	M	c.427G>T	p.(Glu143*)	c.564G>C	p.(Trp188Cys)	Robson et al. 2010	31725702
GC13568	MEH042	UK	M	c.1016 1024delACCTGGTGG	p.(Asp339 Val341del)	c.1016 1024del	p.(Asp339 Val341del)	Robson et al. 2010	31725702
GC13568	MEH043	UK	F	c.1016 1024delACCTGGTGG	p.(Asp339 Val341del)	c.1016 1024del	p.(Asp339 Val341del)	Robson et al. 2010	31725702
GC25878	MEH044	UK	M	c.782C>A	p.(Ala261Asp)	c.782C>A	p.(Ala261Asp)		
GC14604	MEH045	UK	F	c.1316C>T	p.(Thr439Ile)	c.1316C>T	p.(Thr439Ile)		
GC20796	MEH046	UK	M	c.433C>T	p.(Gln145*)	c.1381G>A	p.(Gly461Arg)		
GC20796	MEH047	UK	M	c.433C>T	p.(Gln145*)	c.1381G>A	p.(Gly461Arg)		
GC19863	MEH048	UK	F	c.339C>A	p.(Cys113*)	c.1096del	p.(Val366Trpfs*88)		
RCD 307	RCD307-16334	Germany	M	c.19 1356+9571delinsCATTTG	p.?	c.19 1356+9571delinsCATTTG	p.?	Wissinger et al. 2011	21882291
ZD 395	ZD395-1670	Germany	F	c.1211T>C	p.(Leu404Pro)	NC 000009.11:g.2667638 2747341del	Whole Gene Deletion	Wissinger et al. 2011	21882291
ZD 395	ZD395-7006	Germany	M	c.1211T>C	p.(Leu404Pro)	NC 000009.11:g.2667638 2747341del	Whole Gene Deletion	Wissinger et al. 2011	21882291
ZD 599	ZD599-29894	Germany	F	c.1186G>T	p.(Gly396*)	c.(? -214) 1356+?	Exon 1 Del		
ZD 170	ZD170-11323	Germany	M	c.778A>T	p.(Lys260*)	c.859C>T	p.(Gln287*)	Wissinger et al. 2008	18235024
ZD 170	ZD170-16515	Germany	M	c.778A>T	p.(Lys260*)	c.859C>T	p.(Gln287*)	Wissinger et al. 2008	18235024
ZD 144	ZD144-10240	Germany	F	c.8 11delAACA	p.(Lys3Argfs*96)	c.15 20delinsA	p.(Ser5Argfs*16)	Wissinger et al. 2008	18235024
BD 27	BD27-7318	Germany	M	c.339C>A	p.(Cys113*)	c.442G>T	p.(Glu148*)	Zobor et al. 2012	23077521
BD 27	BD27-7320	Germany	F	c.339C>A	p.(Cys113*)	c.442G>T	p.(Glu148*)	Zobor et al. 2012	23077521
ZD 430	ZD430-21549	Germany	M	c.1356+3 +6delGAGT	p.?	c.1356+3 +6delGAGT	p.?		
ZD 546	ZD546-25834	Germany	M	c.442G>A	p.(Glu148Lys)	c.1381G>A	p.(Gly461Arg)		
ZD 539	ZD539-25438	Germany	F	c.442G>T	p.(Glu148*)	c.442G>T	p.(Glu148*)		
ZD 394	ZD394-4660	Germany	M	c.8 11delAACA	p.(Lys3Argfs*96)	c.447 449del	p.(Phe150del)	Zobor et al. 2012	23077521
1389678	UMCA1	Neitherlands	F	c.1381G>T	p.(Gly461*)	c.1381G>T	p.(Gly461*)		
6315911	UMCA2	Neitherlands	M	c.1356+3_+6delGAGT	p.?	c.1356+3 +6delGAGT	p.?		
LUMC1	LUMC001	Neitherlands	M	c.1349G>A	p.(Trp450*)	c.1381G>A	p.(GIy461Arg)		
LUMC1	LUMC002	Neitherlands	F	c.1349G>A	p.(Trp450*)	c.1381G>A	p.(GIy461Arg)		
ERA01	AAHJT01	Neitherlands	F	c.1123G>A	p.(Val375Met)	c.1123G>A	p.(Val375Met)		
ERA02	AAHJT02	Neitherlands	M	c.(? -214) 1356+?	Exon 1 Deletion	c.(? -214) 1356+?	Exon 1 Deletion		
FJD01	FJD01	Spain	M	c.550G>A	p.(Glu184Lys)	c.778 A>T	p.(Lys260*)		
FJD02	FJD02-1	Spain	M	c.778 A>T	p.(Lys260*)	c.778 A>T	p.(Lys260*)		
FJD02	FJD02-2	Spain	M	c.778 A>T	p.(Lys260*)	c.778 A>T	p.(Lys260*)		
CCAD-F1	CCAD1	UAE	F	c.427G>T	p.(Glu143*)	c.427G>T	p.(Glu143*)	Khan et al 2019	31725702
CCAD-F1	CCAD2	UAE	F	c.427G>T	p.(Glu143*)	c.427G>T	p.(Glu143*)	Khan et al 2019	31725702
CCAD-F2	CCAD3	UAE	F	c.427G>T	p.(Glu143*)	c.427G>T	p.(Glu143*)	Khan et al 2019	31725702
CCAD-F3	CCAD4	UAE	F	c.427G>T	p.(Glu143*)	c.427G>T	p.(Glu143*)	Khan et al 2019	31725702
CCAD-F4	CCAD5	UAE	M	c.427G>T	p.(Glu143*)	c.427G>T	p.(Glu143*)	Khan et al 2019	31725702
CCAD-F5	CCAD6	UAE	M	c.427G>T	p.(Glu143*)	c.427G>T	p.(Glu143*)	Khan et al 2019	31725702
CCAD-F6	CCAD7	UAE	F	c.427G>T	p.(Glu143*)	c.427G>T	p.(Glu143*)	Khan et al 2019	31725702
CCAD-F7	CCAD8	UAE	F	c.427G>T	p.(Glu143*)	c.427G>T	p.(Glu143*)	Khan et al 2019	31725702
CCAD-F8	CCAD9	UAE	F	c.427G>T	p.(Glu143*)	c.427G>T	p.(Glu143*)	Khan et al 2019	31725702
CCAD-F9	CCAD10	UAE	M	c.325C>T	p.(Gln109*)	c.325C>T	p.(Gln109*)	Khan et al 2019	31725702
MOL0089	MOL0089-1	Israel	M	c.782C>A	p.(Ala261Asp)	c.782C>A	p.(Ala261Asp)	Zelinger et al 2013	31725702
MOL0673	MOL0673-1	Israel	M	c.758delC	p.(Pro253Hisfs*68)	c.996_997insGC	p.(Ser333Alafs*121)	Zelinger et al 2013	23725738
MOL0673	MOL0673-2	Israel	F	c.758delC	p.(Pro253Hisfs*68)	c.996_997insGC	p.(Ser333Alafs*121)	Zelinger et al 2013	23725738
MOL0726	MOL0726-1	Israel	M	c.411_414delCCTG	p.(Leu138Alafs*72)	c.411_414delCCTG	p.(Leu138Alafs*72)	Zelinger et al 2013	23725738
MOL0726	MOL0726-2	Israel	F	c.411_414delCCTG	p.(Leu138Alafs*72)	c.411_414delCCTG	p.(Leu138Alafs*72)	Zelinger et al 2013	23725738
MOL0736	MOL0736-1	Israel	F	c.958C>T	p.(Arg320Cys)	c.996_997insGC	p.(Ser333Alafs*121)	Zelinger et al 2013	23725738
MOL0736	MOL0736-2	Israel	F	c.958C>T	p.(Arg320Cys)	c.996_997insGC	p.(Ser333Alafs*121)	Zelinger et al 2013	23725738
MOL1153	MOL1153-1	Israel	F	c.996_997insGC	p.(Ser333Alafs*121)	c.996_997insGC	p.(Ser333Alafs*121)	Zelinger et al 2013	23725738
MOL1254	MOL1254-1	Israel	M	c.455A>G	p.(Asp152Gly)	c.455A>G	p.(Asp152Gly)		
MOL1254	MOL1254-2	Israel	M	c.455A>G	p.(Asp152Gly)	c.455A>G	p.(Asp152Gly)		
A1001	A1001-01	Japan	F	c.529T>C	p.(Cys177Arg)	c.1381G>A	p.(Gly461Arg)	Fujinami et al 2013	23885164
A1001	A1001-02	Japan	M	c.529T>C	p.(Cys177Arg)	c.1381G>A	p.(Gly461Arg)	Fujinami et al 2013	23885164
A1002	A1002	Japan	F	c.80 G>A, c.617 G>C	p.(Arg27His); p.(Arg206Pro)	c.80G>A, c.617G>C	p.(Arg27His); p.(Arg206Pro)	Fujinami et al 2013	23885164
A1004	A1004	Japan	F	c.1381G>A	p.(Gly461Arg)	c.931G>C	p.(Gly311Arg)		
A1005	A1005	Japan	F	c.200G>A	p.(Trp67*)	c.520dupG	p.(Leu173fs)	Kutsuma et al 2019	30877594
A1006	A1006	Japan	F	c.931G>C	p.(Gly311Arg)	c.931G>C	p.(Gly311Arg)		
CA-01	HSC 01	Canada	M	c.459_460dupCG	p.(Asp154Alafs*58)	c.459_460dupCG	p.(Asp154Alafs*58)	Vincent et al. 2013	23221069
CA-02	HSC 02	Canada	M	c.1381G>A	p.(Gly461Arg)	c.1381G>A	p.(Gly461Arg)	Vincent et al. 2013	23221069
CA-O3	HSC 03	Canada	M	c.238G>T	p.(Glu80*)	c.238G>T	p.(Glu80*)	Vincent et al. 2013	23221069
CA-03	HSC 04	Canada	M	c.238G>T	p.(Glu80*)	c.238G>T	p.(Glu80*)	Vincent et al. 2013	23221069
CA-04	HSC 05	Canada	M	c.667C>T	p.(Gln223*)	c.1381G>A	p.(Gly461Arg)	Vincent et al. 2013	23221069
CA-05	HSC 06	Canada	F	c.1381G>A	p.(Gly461Arg)	c.1381G>A	p.(Gly461Arg)	Vincent et al. 2013	23221069
CA-06	HSC 07	Canada	F	c.1016_1024delACCTGGTGG	p.(Asp339_Val341del)	c.1016_1024delACCTGGTGG	p.(Asp339_Val341del)	Vincent et al. 2013	23221069
CA-07	HSC 08	Canada	M	c.1381G>A	p.(Gly461Arg)	c.1381G>A	p.(Gly461Arg)		
CA-08	HSC 09	Canada	M	c.8_11delAACA	p.(Lys3Argfs*96)	c.8_11delAACA	p.(Lys3Argfs*96)		
MEE01	MEE01	USA	F	c.721_722delCCinsTA	p.(Pro241*)	NA	NA	Thiagalingam et al. 2007	1700774
MEE02	MEE02	USA	F	c.778A>T	p.(Lys260*)	c.867delC	p.(Ser289fs)	Thiagalingam et al. 2007	1700774
MEE03	MEE03	USA	F	c.778A>T	p.(Lys260*)	c.1637T>C	p.(*546Glnext*?	Thiagalingam et al. 2007	1700774
MEE03	MEE04	USA	F	c.778A>T	p.(Lys260*)	c.1637T>C	p.(*546Glnext*?)	Thiagalingam et al. 2007	1700774
MEE05	MEE05	USA	M	c.1381G>A	p.(Gly461Arg)	c.473T>G	p.(Phe158Cys)		
MEE06	MEE06	USA	M	c.325C>T	p.(Gln109*)	c.325C>T	p.(Gln109*)		
MEE07	MEE07	USA	M	c.1336dupC	p.(His446Profs*53)	c.1336dupC	p.(His446Profs*53)		
2875	CEI-01	USA	F	c.1381G>A	p.(Gly461Arg)	c.638G>C	p.(Arg213Pro)	Thiagalingam et al. 2007	1700774
2672	CEI-02	USA	F	c.1381G>A	p.(Gly461Arg)	c.533C>T	p.(Pro178Leu)	Thiagalingam et al. 2007	1700774
4328	CEI-03	USA	F	c.778A>T	p.(Lys260*)	c.1375G>T	p.(Gly459Cys)		
3013	CEI-04	USA	M	c.1348T>G	p.(Trp450Gly)	c.1348T>G	p.(Trp450Gly)	Thiagalingam et al. 2007	1700774
CEI-P1	CEI-05	USA	F	c.451T>C	p.(Phe151Leu)	c.1381G>A	p.(Gly461Arg)		

†Reference sequence: NM 133497.4
