# Supplementary material for: KCNV2-Associated Retinopathy: Genetics, Electrophysiology, and Clinical Course—KCNV2 Study Group Report 1
Source: Am J Ophthalmol. 2021 May;225:95–107. doi: 10.1016/j.ajo.2020.11.022 (PMC8186730; doi:10.1016/j.ajo.2020.11.022)
Supplement: Supplementary Table 2 [file mmc2.pdf]

Supplementary Table 2: *KCNV2* Variants Identified in Study-117

CHROM	POS†	ID		REF	ALT	most_severe_consequence	af_kaviar	af_gnomad_genomes	af_tommo	af_krgdb	af_hgvd	gene_symbol	hgvsc	hgvsp	dann	cadd_phred	Identified Alleles in Study-117 (n=)	Frequency (%)	ACMG prediction	Factor 1	Factor 2	Factor 3	Factor 4	Factor 5	Factor 6
9	2717740	KCNV2:c.1A>G	KCNV2:p.Met1?	A	G	start_lost	0.0000064	0.0000000	0	0	0	KCNV2	ENST00000382082.3:c.1A>G	ENSP00000371514.3:p.Met1?	0.973	10.35	1	0.43%	Likely Pathogenic	PVS1	PM2	PP3	PP4		
9	2717744	KCNV2:c.8_11delAACA	KCNV2:p.Lys3Argfs*96	TCAAA	T	frameshift_variant	0	0	0	0	0	KCNV2	ENST00000382082.3:c.8_11delAACA	ENSP00000371514.3:p.Lys3ArgfsTer96		24.3	5	2.17%	Pathogenic	PVS1	PM2	PP3	PP4	PP5	
9	2717746	KCNV2:c.7A>T	KCNV2:p.Lys3*	A	T	stop_gained	0	0.0000000	0	0	0	KCNV2	ENST00000382082.3:c.7A>T	ENSP00000371514.3:p.Lys3Ter	0.994	35	2	0.87%	Pathogenic	PVS1	PM2	PP3	PP4		
9	2717754	KCNV2:c.15_20delinsA	KCNV2:p.Ser5Argfs*16	TGAGAG	A	frameshift_variant	0	0.0000000	0	0	0	KCNV2	ENST00000382082.3:c.15_20delinsA	ENSP00000371514.3:p.Ser5ArgfsTer16		27.4	1	0.43%	Pathogenic	PVS1	PM2	PP3	PP4		
9	2717758	KCNV2:c.19_1356+9571delinsCATTTG	KCNV2:p.?	AGGAGACGGTCCTGC	CATTTG	deletion	0	0.0000000	0	0	0	KCNV2	ENST00000382082.3:c.19_1356+9571delinsCATTTG			NA	2	0.87%	Pathogenic	PVS1	PM2	PP3	PP4		
9	2717819	‡KCNV2:c.80G>A	KCNV2:p.Arg27His	G	A	missense_variant	0.0011575	0.0009549	0.0135	0	0.01217	KCNV2	ENST00000382082.3:c.80G>A	ENSP00000371514.3:p.Arg27His	0.999	23.4	2‡	0.87%	Benign	PP2	BS1	BS2	BP4		
9	2717939	KCNV2:c.200G>A	KCNV2:p.Trp67*	G	A	stop_gained	0	0	0	0	0	KCNV2	ENST00000382082.3:c.200G>A	ENSP00000371514.3:p.Trp67Ter	0.987	27.6	1	0.43%	Pathogenic	PVS1	PM2	PP4	PP5		
9	2717962	KCNV2:c.224_230delACCAGCA	KCNV2:p.Asp75Glyfs*23	GACCAGCA	G	frameshift_variant	0	0.0000000	0	0	0	KCNV2	ENST00000382082.3:c.224_230delACCAGCA	ENSP00000371514.3:p.Asp75Glyfs*23		22.9	2	0.87%	Pathogenic	PVS1	PM2	PP4			
9	2717977	KCNV2:c.238G>T	KCNV2:p.Glu80*	G	T	stop_gained	0	0.0000000	0	0	0	KCNV2	ENST00000382082.3:c.238G>T	ENSP00000371514.3:p.Glu80Ter	0.992	29.1	4	1.74%	Pathogenic	PVS1	PM2	PP4			
9	2718064	KCNV2:c.325C>T	KCNV2:p.Gln109*	C	T	stop_gained	0	0.0000000	0	0	0	KCNV2	ENST00000382082.3:c.325C>T	ENSP00000371514.3:p.Gln109Ter	0.997	35	10	4.35%	Pathogenic	PVS1	PM2	PP3	PP4		
9	2718078	KCNV2:c.339C>A	KCNV2:p.Cys113*	C	A	stop_gained	0.0000064	0.0000318	0	0	0	KCNV2	ENST00000382082.3:c.339C>A	ENSP00000371514.3:p.Cys113Ter	0.974	26.1	3	1.30%	Pathogenic	PVS1	PM2	PP4	PP5	BP4	
9	2718116	KCNV2:c.377T>A	KCNV2:p.Leu126Gln	T	A	missense_variant	0	0.0000000	0	0	0	KCNV2	ENST00000382082.3:c.377T>A	ENSP00000371514.3:p.Leu126Gln	0.995	23.7	1	0.43%	Likely Pathogenic	PM2	PP2	PP3	PP4	PP5	
9	2718149	KCNV2:c.411_414delCCTG	KCNV2:p.Leu138Alafs*72	CAGGC	C	frameshift_variant	0	0	0	0	0	KCNV2	ENST00000382082.3:c.411_414delCCTG	ENSP00000371514.3:p.Leu138AlafsTer72		0.448	4	1.74%	Pathogenic	PVS1	PM2	PP3	PP4	PP5	
9	2718156	KCNV2:c.417C>A	KCNV2:p.Cys139*	C	A	stop_gained	0.0000064	0.0000000	0	0	0	KCNV2	ENST00000382082.3:c.417C>A	ENSP00000371514.3:p.Cys139Ter	0.993	35	2	0.87%	Pathogenic	PVS1	PM2	PP4	PP5		
9	2718166	KCNV2:c.427G>T	KCNV2:p.Glu143*	G	T	stop_gained	0.0000064	0.0000000	0	0	0	KCNV2	ENST00000382082.3:c.427G>T	ENSP00000371514.3:p.Glu143Ter	0.997	37	29	12.61%	Pathogenic	PVS1	PM2	PP3	PP4	PP5	
9	2718172	KCNV2:c.433C>T	KCNV2:p.Gln145*	C	T	stop_gained	0.0000064	0.0000000	0	0	0	KCNV2	ENST00000382082.3:c.433C>T	ENSP00000371514.3:p.Gln145Ter	0.994	28.9	3	1.30%	Pathogenic	PVS1	PM2	PP4			
9	2718181	KCNV2:c.442G>A	KCNV2:p.Glu148Lys	G	A	missense_variant	0.0000064	0.0000318	0	0	0	KCNV2	ENST00000382082.3:c.442G>A	ENSP00000371514.3:p.Glu148Lys	0.999	27.5	1	0.43%	Likely Pathogenic	PM2	PP2	PP3	PP4		
9	2718181	KCNV2:c.442G>T	KCNV2:p.Glu148*	G	T	stop_gained	0.0000064	0.0000000	0.0001	0	0	KCNV2	ENST00000382082.3:c.442G>T	ENSP00000371514.3:p.Glu148Ter	0.998	38	5	2.17%	Pathogenic	PVS1	PM2	PP3	PP4	PP5	
9	2718183	KCNV2:c.445_446delTA	KCNV2:p.Tyr149LeufsTer222	ATA	A	frameshift_variant	0	0	0	0	0	KCNV2	ENST00000382082.3:c.445_446del	ENSP00000371514.3:p.Tyr149LeufsTer222		32	2	0.87%	Pathogenic	PVS1	PM2	PP3	PP4		
9	2718185	KCNV2:c.447_449del	KCNV2:p.Phe151del	ACTT	A	inframe_deletion	0.0000064	0	0	0	0	KCNV2	ENST00000382082.3:c.451_453del	ENSP00000371514.3:p.Phe151del		14.92	1	0.43%	Likely Pathogenic	PM2	PM4	PP3	PP4		
9	2718190	KCNV2:c.451T>C	KCNV2:p.Phe151Val	T	C	missense_variant	0.0000064	0.0000000	0	0	0	KCNV2	ENST00000382082.3:c.451T>C	ENSP00000371514.3:p.Phe151Leu	0.999	25.8	4	1.74%	Likely Pathogenic	PM2	PP2	PP3	PP4		
9	2718194	KCNV2:c.455A>G	KCNV2:p.Asp152Gly	A	G	missense_variant	0	0.0000000	0	0	0	KCNV2	ENST00000382082.3:c.455A>G	ENSP00000371514.3:p.Asp152Gly	0.998	28.3	4	1.74%	Likely Pathogenic	PM2	PP3	PP4	PP5		
9	2718198	KCNV2:c.459_460dupCG	KCNV2:p.Asp154Alafs*58	CG	CGCG	frameshift_variant	0	0	0	0	0	KCNV2	ENST00000382082.3:c.459_460dupCG	ENSP00000371514.3:p.Asp154Alafs*58		34	2	0.87%	Pathogenic	PVS1	PM2	PP3	PP4		
9	2718212	KCNV2:c.473T>G	KCNV2:p.Phe158Cys	T	G	missense_variant	0	0.0000000	0	0	0	KCNV2	ENST00000382082.3:c.473T>G	ENSP00000371514.3:p.Phe158Cys	0.995	26.3	1	0.43%	Likely Pathogenic	PM1	PM2	PP3	PP4		
9	2718223	KCNV2:c.484T>A	KCNV2:p.Tyr162Asn	T	A	missense_variant	0	0.0000000	0	0	0	KCNV2	ENST00000382082.3:c.484T>A	ENSP00000371514.3:p.Tyr162Asn	0.989875	23.2	1	0.43%	Likely Pathogenic	PM1	PM2	PP3	PP4		
9	2718233	KCNV2:c.494A>G	KCNV2:p.Tyr165Cys	A	G	missense_variant	0.0000064	0.0000000	0	0	0	KCNV2	ENST00000382082.3:c.494A>G	ENSP00000371514.3:p.Tyr165Cys	0.998	25.9	1	0.43%	Likely Pathogenic	PM1	PM2	PP3	PP4		
9	2718259	KCNV2:c.520dupG	KCNV2:p.Asp174Glyfs*198	G	GG	frameshift_variant	0	0.0000000	0	0	0	KCNV2	ENST00000382082.3:c.520dupG	ENSP00000371514.3::p.Asp174Glyfs*198		27.3	1	0.43%	Pathogenic	PVS1	PM2	PP3	PP4	PP5	
9	2718268	KCNV2:c.529T>C	KCNV2:p.Cys177Arg	T	C	missense_variant	0.0000064	0.0000000	0.0003	0	0	KCNV2	ENST00000382082.3:c.529T>C	ENSP00000371514.3:p.Cys177Arg	0.99	23.1	2	0.87%	Likely Pathogenic	PM2	PP2	PM5	PP3	PP4	PP5
9	2718272	KCNV2:c.533C>T	KCNV2:p.Pro178Leu	C	T	missense_variant	0.0000193	0.0000000	0	0	0	KCNV2	ENST00000382082.3:c.533C>T	ENSP00000371514.3:p.Pro178Leu	0.997	23.7	1	0.43%	Likely Pathogenic	PM1	PM2	PP3	PP4		
9	2718289	KCNV2:c.550G>A	KCNV2:p.Glu184Lys	G	A	missense_variant	0	0.0000000	0	0	0	KCNV2	ENST00000382082.3:c.550G>A	ENSP00000371514.3:p.Glu184Lys	0.999	27.9	1	0.43%	Likely Pathogenic	PM1	PM2	PP3	PP4		
9	2718301	KCNV2:c.562T>A	KCNV2:p.Trp188Arg	T	A	missense_variant	0	0.0000000	0	0	0	KCNV2	ENST00000382082.3:c.562T>A	ENSP00000371514.3:p.Trp188Arg	0.995	26	1	0.43%	Likely Pathogenic	PM2	PM5	PP3	PP4		
9	2718303	KCNV2:c.564G>C	KCNV2:p.Trp188Cys	G	C	missense_variant	0.0000129	0.0000000	0	0	0	KCNV2	ENST00000382082.3:c.564G>C	ENSP00000371514.3:p.Trp188Cys	0.992	26	1	0.43%	Likely Pathogenic	PM2	PM5	PP3	PP4		
9	2718304	KCNV2:c.566delG	KCNV2:p.Gly189Alafs*22	GG	G	frameshift_variant	0	0	0	0	0	KCNV2	ENST00000382082.3:c.566del	ENSP00000371514.3:p.Gly189AlafsTer22		33	4	1.74%	Pathogenic	PVS1	PM2	PP3	PP4		
9	2718356	‡KCNV2:c.617G>C	KCNV2:p.Arg206Pro	G	C	missense_variant	0	0.0000000	0.0001	0	0	KCNV2	ENST00000382082.3:c.617G>C	ENSP00000371514.3:p.Arg206Pro	0.997	29.1		0.00%	Uncertain Significance	PM2	PP3	PP4			
9	2718377	KCNV2:c.638G>C	KCNV2:p.Arg213Pro	G	C	missense_variant	0.0000965	0.0000637	0	0	0	KCNV2	ENST00000382082.3:c.638G>C	ENSP00000371514.3:p.Arg213Pro	0.957	23	1	0.43%	Uncertain Significance	PM2	PP2	PP4	BP4		
9	2718406	KCNV2:c.667C>T	KCNV2:p.Gln223*	C	T	stop_gained	0	0.0000000	0	0	0	KCNV2	ENST00000382082.3:c.667C>T	ENSP00000371514.3:p.Gln223Ter	0.997	36	1	0.43%	Pathogenic	PVS1	PM2	PP3	PP4		
9	2718460	KCNV2:c.721_722delCCinsTA	KCNV2:p.Pro241*	CC	TA	stop_gained	0	0.0000000	0	0	0	KCNV2	ENST00000382082.3:c.721_722delinsTA	ENSP00000371514.3:p.Pro241Ter		36	1	0.43%	Pathogenic	PVS1	PM2	PP3	PP4	PP5	
9	2718496	KCNV2:c.758delC	p.Pro253Hisfs*68	C	GC	frameshift_variant	0	0	0	0	0	KCNV2	ENST00000382082.3:c.756_757insG	ENSP00000371514.3:p.Pro253AlafsTer119		34	3	1.30%	Pathogenic	PVS1	PM2	PP3	PP4	PP5	
9	2718505	KCNV2:c.766T>G	KCNV2:p.Ser256Ala	T	G	missense_variant	0	0.0000000	0	0	0	KCNV2	ENST00000382082.3:c.766T>G	ENSP00000371514.3:p.Ser256Ala	0.987	24	1	0.43%	Likely Pathogenic	PM2	PM5	PP3	PP4		
9	2718506	KCNV2:c.767C>G	KCNV2:p.Ser256Trp	C	G	missense_variant	0.0000064	0.0000000	0	0	0	KCNV2	ENST00000382082.3:c.767C>G	ENSP00000371514.3:p.Ser256Trp	0.991	27.3	2	0.87%	Likely Pathogenic	PM2	PP3	PP4	PP5		
9	2718514	KCNV2:c.775G>A	KCNV2:p.Ala259Thr	G	A	missense_variant	0	0.0000000	0	0	0	KCNV2	ENST00000382082.3:c.775G>A	ENSP00000371514.3:p.Ala259Thr	0.998	26.9	2	0.87%	Likely Pathogenic	PM2	PM5	PP3	PP4		
9	2718517	KCNV2:c.778A>T	KCNV2:p.Lys260*	A	T	stop_gained	0.0000064	0.0000318	0	0	0	KCNV2	ENST00000382082.3:c.778A>T	ENSP00000371514.3:p.Lys260Ter	0.996	39	17	7.39%	Likely Pathogenic	PM2	PP3	PP4	PP5		
9	2718521	KCNV2:c.782C>A	KCNV2:p.Ala261Asp	C	A	missense_variant	0.0000129	0.0000000	0	0	0	KCNV2	ENST00000382082.3:c.782C>A	ENSP00000371514.3:p.Ala261Asp	0.988	23	4	1.74%	Pathogenic	PVS1	PM2	PP3	PP4	PP5	
9	2718598	KCNV2:c.859C>T	KCNV2:p.Gln287*	C	T	stop_gained	0.0000257	0.0000000	0	0	0	KCNV2	ENST00000382082.3:c.859C>T	ENSP00000371514.3:p.Gln287Ter	0.997	36	2	0.87%	Likely Pathogenic	PM2	PP3	PP4	PP5		
9	2718604	KCNV2:c.866delC	p.Ser289Trpfs*33	TC	T	frameshift_variant	0	0	0	0	0	KCNV2	ENST00000382082.3:c.870del	ENSP00000371514.3:p.Gln291ArgfsTer31		12.99	1	0.43%	Likely Pathogenic	PVS1	PM2	PP4	BP4		
9	2718605	KCNV2:c.866C>A	KCNV2:p.Ser289*	C	A	stop_gained	0.0000064	0.0000955	0	0	0	KCNV2	ENST00000382082.3:c.866C>A	ENSP00000371514.3:p.Ser289Ter	0.969	32	1	0.43%	Pathogenic	PVS1	PM2	PP3	PP4		
9	2718629	KCNV2:c.874_889dupGGCGAGGGCGGCCCAG	KCNV2:p.Asp297Glyfs*80	A	AGGCGAGGGCGGCCCAG	frameshift_variant	0	0	0	0	0	KCNV2	ENST00000382082.3:c.890_891insGGCGAGGGCGGCCCAG	ENSP00000371514.3:p.Asp297GlufsTer80		22.8	1	0.43%	Pathogenic	PVS1	PM2	PP4	PP5		
9	2718655	KCNV2:c.916G>T	KCNV2:p.Gly306*	G	T	stop_gained	0.0000064	0.0000000	0	0	0	KCNV2	ENST00000382082.3:c.916G>T	ENSP00000371514.3:p.Glu306Ter	0.998	42	2	0.87%	Pathogenic	PVS1	PM2	PP3	PP4		
9	2718670	KCNV2:c.931G>C	KCNV2:p.Gly311Arg	G	C	missense_variant	0.0000064	0.0000000	0.0008	0.000909	0.000414	KCNV2	ENST00000382082.3:c.931G>C	ENSP00000371514.3:p.Gly311Arg	0.994	22.5	3	1.30%	Pathogenic	PVS1	PM2	PP3	PP4	PP5	
9	2718697	KCNV2:c.958C>T	KCNV2:p.Arg320Cys	C	T	missense_variant	0.0000129	0.0000318	0	0	0	KCNV2	ENST00000382082.3:c.958C>T	ENSP00000371514.3:p.Arg320Cys	0.999	32	2	0.87%	Uncertain Significance	PM2	PP3	PP4			
9	2718729	KCNV2:c.996_997insGC	KCNV2:p.Ser333Alafs*121	C	CGC	frameshift_variant	0	0	0	0	0	KCNV2	ENST00000382082.3:c.995_996dup	ENSP00000371514.3:p.Ser333AlafsTer122	0	35	6	2.61%	Likely Pathogenic	PM2	PM5	PP3	PP4	PP5	
9	2718753	KCNV2:c.1016_1024delACCTGGTGG	p.Asp339_Val341del	GGACCTGGTGGC	G	frameshift_variant	0	0	0	0	0	KCNV2	ENST00000382082.3:c.1015_1025del	ENSP00000371514.3:p.Asp339HisfsTer29	0	35	6	2.61%	Pathogenic	PVS1	PM2	PP3	PP4		
9	2718833	KCNV2:c.1096del	KCNV2:p.Val366TrpfsTer88	CG	C	frameshift_variant	0	0	0	0	0	KCNV2	ENST00000382082.3:c.1096del	ENSP00000371514.3:p.Val366TrpfsTer88		14.19	1	0.43%	Pathogenic	PVS1	PM2	PP3	PP4		
9	2718848	KCNV2:c.1110_1128del	KCNV2:p.Lys371AlafsTer77	GTAAGGTGGGTCAGC	G	frameshift_variant	0	0	0	0	0	KCNV2	ENST00000382082.3:c.1110_1128del	ENSP00000371514.3:p.Lys371AlafsTer77		35	2	0.87%	Pathogenic	PVS1	PM2	PP4			
9	2718862	KCNV2:c.1123G>A	KCNV2:p.Val375Met	G	A	missense_variant	0.0003858	3.18E-05	0	0	0	KCNV2	ENST00000382082.3:c.1123G>A	ENSP00000371514.3:p.Val375Met	0.999	26.6	2	0.87%	Pathogenic	PVS1	PM2	PP3	PP4		
9	2718925	KCNV2:c.1186G>T	KCNV2:p.Gly396*	G	T	stop_gained	0.0000064	0.0000000	0	0	0	KCNV2	ENST00000382082.3:c.1186G>T	ENSP00000371514.3:p.Gly396Ter	0.992	42	1	0.43%	Uncertain Significance	PM2	PP3	PP4			
9	2718936	KCNV2:c.1199delT	KCNV2:p.Phe400Serfs*54	CT	C	frameshift_variant	0.0000129	0	0	0	0	KCNV2	ENST00000382082.3:c.1199del	ENSP00000371514.3:p.Phe400SerfsTer54		32	2	0.87%	Pathogenic	PVS1	PM2	PP3	PP4		
9	2718950	KCNV2:c.1211T>C	KCNV2:p.Leu404Pro	T	C	missense_variant	0	0.0000000	0	0	0	KCNV2	ENST00000382082.3:c.1211T>C	ENSP00000371514.3:p.Leu404Pro	0.999	26.4	2	0.87%	Pathogenic	PVS1	PM2	PP3	PP4		
9	2719055	KCNV2:c.1316C>T	KCNV2:p.Thr439Ile	C	T	missense_variant	0	0.0000000	0	0	0	KCNV2	ENST00000382082.3:c.1316C>T	ENSP00000371514.3:p.Thr439Ile	0.999	27.1	2	0.87%	Likely Pathogenic	PM2	PM3	PP3	PP4		
9	2719070	KCNV2:c.1336dupC	KCNV2:p.His446Profs*53	T	TC	frameshift_variant	0	0	0	0	0	KCNV2	ENST00000382082.3:c.1336dup	ENSP00000371514.3:p.His446ProfsTer53	0	35	2	0.87%	Uncertain Significance	PM2	PP3	PP4			
9	2719087	KCNV2:c.1348T>G	KCNV2:p.Trp450Gly	T	G	missense_variant	0.0000064	0.0000000	0	0	0	KCNV2	ENST00000382082.3:c.1348T>G	ENSP00000371514.3:p.Trp450Gly	0.977	26.7	2	0.87%	Pathogenic	PVS1	PM2	PP3	PP4		
9	2719088	KCNV2:c.1349G>A	KCNV2:p.Trp450*	G	A	stop_gained	0	0.0000000	0	0	0	KCNV2	ENST00000382082.3:c.1349G>A	ENSP00000371514.3:p.Trp450Ter	0.997	44	2	0.87%	Likely Pathogenic	PM2	PM5	PP3	PP4		
9	2719097	KCNV2:c.1356+3_+6delGAGT	p.?	TGAGT	T	splice_region_variant	0.0000064	0	0	0	0	KCNV2	ENST00000382082.3:c.1356+3_1356+6del			24.6	4	1.74%	Pathogenic	PVS1	PM2	PP3	PP4		
9	2729464	KCNV2:c.1375G>T	KCNV2:p.Gly459Cys	G	T	missense_variant	0	0.0000000	0	0	0	KCNV2	ENST00000382082.3:c.1375G>T	ENSP00000371514.3:p.Gly459Cys	0.996	34	1	0.43%	Likely Pathogenic	PM2	PM5	PP3	PP4		
9	2729465	KCNV2:c.1376G>A	KCNV2:p.Gly459Asp	G	A	missense_variant	0.0000064	0.0000000	0	0	0	KCNV2	ENST00000382082.3:c.1376G>A	ENSP00000371514.3:p.Gly459Asp	0.998	33	2	0.87%	Likely Pathogenic	PM1	PM2	PP3	PP4	PP5	
9	2729470	KCNV2:c.1381G>A	KCNV2:p.Gly461Arg	G	A	missense_variant	0.0001479	0.0000637	0	0	0.000421	KCNV2	ENST00000382082.3:c.1381G>A	ENSP00000371514.3:p.Gly461Arg	0.999	34	26	11.30%	Pathogenic	PM1	PM2	PP3	PP4	PP5	
9	2729470	KCNV2:c.1381G>T	KCNV2:p.Gly461*	G	T	stop_gained	0.0000064	0.0000318	0	0	0	KCNV2	ENST00000382082.3:c.1381G>T	ENSP00000371514.3:p.Gly461Ter	0.994	52	4	1.74%	Pathogenic	PVS1	PM2	PP3	PP4		
9	2729493	KCNV2:c.1404delC	KCNV2:p.Leu469Trpfs*35	AC	A	frameshift_variant	0	0	0	0	0	KCNV2	ENST00000382082.3:c.1405del	ENSP00000371514.3:p.Leu469TrpfsTer35		35	2	0.87%	Pathogenic	PVS1	PM2	PP3	PP4		
9	2729725	KCNV2:c.1636T>C	KCNV2:p.*546Glnext*?	T	C	stop_lost	0	0	0	0	0	KCNV2	ENST00000382082.3:c.1636T>C	ENSP00000371514.3:p.*546Glnext*?	0.791	13.8	2	0.87%	Uncertain Significance	PM2	PM4	PP4	BP4		
9	2729727	KCNV2:c.1638G>T	KNCV2:p.*546Tyrext*60	G	T	stop_lost	0	0.0000000	0	0	0	KCNV2	ENST00000382082.3:c.1638G>T	ENSP00000371514.3:p.*546Tyrext*60	0.642	0.002	1	0.43%	Uncertain Significance	PM2	PM4	PP4	BP4		
9	NA	arr(GRCh37)9p.24.2(2670960_2783870)x0	Whole Gene Del			Whole Gene Deletion											2	0.87%	NA						
9	NA	g.2657638_2737340del	Whole Gene Del			Whole Gene Deletion											2	0.87%	NA						
9	NA	c.(?_-214)_1356+?	Exon 1 Del			Exon Deletion											2	0.87%	NA						
9	NA	c.1-?_1356+?del	Exon 1 Del			Exon Deletion											1	0.43%	NA						
9	NA	c.1357-?_1638+?del	Exon 2 Del			Exon Deletion											2	0.87%	NA						

† Reference assembly: GRCh37/hg19

‡ Complex allele c.80 G>A, c.617 G>C : The p.Arg27His is in 2% of east asian alleles while the second is absent from gnomad.

PVS1 Null variant (nonsense, frameshift, canonical +/−1 or 2 splice sites, initiationcodon, single or multi-exon deletion) in a gene where loss of function (LOF)is a known mechanism of disease

PS1 Same amino acid change as a previously established pathogenic variantregardless of nucleotide change

PS2 De novo (both maternity and paternity confirmed) in a patient with thedisease and no family history

PM1 Located in a mutational hot spot and/or critical and well-establishedfunctional domain

PM2 Absent from controls (or at extremely low frequency if recessive)in Exome Sequencing Project, 1000 Genomes or ExAC

PM3 For recessive disorders, detected in trans with a pathogenic variant

PM5 Novel missense change at an amino acid residue where a differentmissense change determined to be pathogenic has been seen before

PP2 Missense variant in a gene that has a low rate of benign missense variationand where missense variants are a common mechanism of disease

PP3 Multiple lines of computational evidence support a deleterious effect onthe gene or gene product (conservation, evolutionary, splicing impact, etc)

PP4 Patient’s phenotype or family history is highly specific for a disease with asingle genetic etiology

PP5 Reputable source recently reports variant as pathogenic but the evidence is not available to the laboratory to perform an independent evaluation

BP4 Multiple lines of computational evidence suggest no impact on gene orgene product (conservation, evolutionary, splicing impact, etc)

BP6 Reputable source recently reports variant as benign but the evidence is notavailable to the laboratory to perform an independent evaluation

RefSeq reference: NM_133497.4
