# Supplementary material for: KCNV2-Associated Retinopathy: Genetics, Electrophysiology, and Clinical Course—KCNV2 Study Group Report 1
Source: Am J Ophthalmol. 2021 May;225:95–107. doi: 10.1016/j.ajo.2020.11.022 (PMC8186730; doi:10.1016/j.ajo.2020.11.022)
Supplement: Supplementary Table 3 [file mmc3.pdf]

Supplementary Table 3: Novel *KCNV2* variants Identified in Study-117

Position	HGVSc	HGVSp
2717740	c.1A>G	p.Met1?
2717754	c.15_20delinsA	p.Ser5Argfs*16
2718156	c.417C>A	p.Cys139*
2718172	c.433C>T	p.Gln145*
2718181	c.442G>A	p.Glu148Lys
2718183	c.445_446delTA	p.Tyr149Leufs*222
2718185	c.447_449del	p.Phe150del
2718194	c.455A>G	p.Asp152Gly
2718233	c.494A>G	p.Tyr165Cys
2718301	c.562T>A	p.Trp188Arg
2718304	c.566delG	p.Gly189Alafs*22
2718505	c.766T>G	p.Ser256Ala
2718605	c.866C>A	p.Ser289*
2718629	c.874_889dupGGCGAGGGCGGCCCAG	p.Asp297Glyfs*80
2718670	c.G931C	p.Gly311Arg
2718753	c.1016_1024delACCTGGTGG	p.Asp339_Val341del
2718833	c.1096del	p.Val366Trpfs*88
2718848	c.1110_1128del	p.Lys371Alafs*77
2718925	c.1186G>T	p.Gly396*
2719070	c.1336dupC	p.His446Profs*53
2719088	c.1349G>A	p.Trp450*
2719097	c.1356+3_+6delGAGT	p.?
2729464	c.1375G>T	p.Gly459Cys
2729725	c.1636T>C	p.X546GlnextX61
2718223	c.484T>A	p.Tyr162Asn
2717960	KCNV2:c.224_230delACCAGCA	KCNV2:p.Asp75Glyfs*23
NA	c.(?_-214)_1356+?	Exon 1 Del
NA	c.1357-?_1638+?del	Exon 2 Del

HGVS: Human Genome Variation Society nomenclature
